# Genome-wide insights into adaptive divergence, historical demography, and habitat suitability of *Ptychobarbus Kaznakovi* and *P. leptosomus*

**DOI:** 10.1186/s12864-026-12664-4

**Published:** 2026-02-25

**Authors:** Taiming Yan, Ping Chen, Huiling Wang, Mengna Chang, Qipeng Fu, Wenjie Luo, Fei Liu, Junjie Huang, Wenxiang Ding, Kuo Gao, Lin Wen, Jinxing Xiong, Haochen Wang, Rukui Zeng, Ziting Tang, Zhi He, Deying Yang

**Affiliations:** 1 Fisheries College, Sichuan Agricultural University, 211 # Huimin Road, Chengdu, China; 2 Yalong River Hydropower Development Company, Ltd, Chengdu, China

**Keywords:** Ptychobarbus kaznakovi, P. leptosomus, Historical population dynamics, Population structure, Single-nucleotide polymorphism (SNP), Whole-genome level

## Abstract

**Background:**

Our previous study from 2024 indicated that *Ptychobarbus leptosomus* is a new species found only in the Yalong River (the largest tributary of the Jinsha River). *P. leptosomus* was historically classified as *P. kaznakovi*, which lives in the Jinsha River. To date, the evolutionary history and population dynamics of *P. leptosomus* and *P. kaznakovi* have not been reported. In our study, both species have similar morphologies, which may reflect gene flow between the two species. Genotyping-by-sequencing (GBS) technology was utilized to acquire whole-genome single-nucleotide polymorphism (SNP) markers, which were subsequently used to assess population structure, population dynamics, and adaptive differentiation.

**Results:**

Phylogenetic and population structural analyses based on SNPs indicated that *P. leptosomus* is an independent Picea species. Additionally, *P. kaznakov* is more closely related to *P. chungtienensis*, which is consistent with its geographic distribution. The obvious gene flow from *P. kaznakovi* and *P. chungtienensis* branches to *P. dipogon* was detected. Historical population dynamics analysis revealed that tectonic events in the Shaluli Mountains and the Quaternary climate oscillation had important impacts on the current distribution patterns of the two species, which experienced similar population contraction and expansion processes. Local adaptation promoted differentiation between *P. leptosomus* and *P. kaznakov.* Genotype and environment association analysis revealed that 35,654 SNPs were related to environmental factors, mainly related to adaptation to precipitation seasonality and temperature seasonality. Selective elimination analysis revealed that the selected genes were enriched mainly in glycan biosynthesis and metabolism and growth hormone synthesis, secretion, and action (genes such as *glycine decarboxylase (gldc*), *cyp51*,* igf-1*, and *tnf-α*), which can help *P. leptosomus* and *P. kaznakov* adapt better to the water environment of the high mountains and valleys in the Shaluli Mountains.

**Conclusions:**

This study emphasizes the significant role of geological and environmental changes in shaping the population history and evolutionary processes of *P. kaznakovi* and *P. leptosomus*, and deepens our understanding of the species classification of *Ptychobarbus* and provides a basis for future species protection.

**Supplementary Information:**

The online version contains supplementary material available at 10.1186/s12864-026-12664-4.

## Background

Complex geological events (such as river capture) and climate change-induced geographical isolation are the major drivers shaping the current genetic diversity and structural patterns of many biotas [1, 2]. The complex ecological environment of the mountainous regions in Southwest China, with their geological and climatic variations, has led to intricate changes in landscapes and waterways, resulting in the isolation and independent evolution of aquatic species [2]. The gene flow between geographically or ecologically isolated populations is often highly restricted. Unique adaptive genetic characteristics may occur in isolated populations exposed to different food, landscape and hydrological conditions. The Shaluli Mountains, situated between the Jinsha River and the Yalong River in the southeastern Tibetan Plateau, constitute the largest mountain range in the Hengduan Mountains region. Formed as part of the uplift of the Tibetan Plateau, the Shaluli Mountains have played a key role in past glacial events in the southeastern plateau and continue to play a crucial role in the origin and differentiation of fish species within the Jinsha River Basin [3, 4].

Fish possess strong environmental adaptability, enabling them to respond rapidly to environmental changes. Through adaptive evolutionary processes, they can ultimately evolve into new species. Compared with other vertebrates, the geographical distribution of fish is more tightly linked to aquatic environments and is strictly constrained by river system structures. Furthermore, the population structure and genetic differentiation of fish are more susceptible to the influence of geological events. The genus *Ptychobarbus* comprises specialized-grade *Schizothorax* fish whose evolutionary history is closely tied to the uplift of the Tibetan Plateau, having formed and evolved gradually within this geological context. To date, *Ptychobarbus* consists of five species, but only *P. leptosomus* and *P. kaznakovi* are distributed within the Shaluli Mountains region [5]. *P. leptosomus* was identified as a new species by Zhang et al. in 2019 and is restricted to the Yalong River system [6], whereas *P. kaznakovi* is widely distributed in the Jinsha River system [7]. Originating on the Qinghai‒Tibet Plateau (QTP), the Jinsha River flows southeast through mountainous terrain, receiving several major tributaries within western Sichuan; its principal tributary, the Yalong River, drains a parallel basin separated from the main Jinsha channel by the Shaluli Mountains. These two river systems converge in Panzhihua City, Sichuan Province (elevation 980 m). Across the broader Jinsha River basin, *P. leptosomus* and *P. kaznakovi* inhabit diverse aquatic environments. Their distribution encompasses both river drainages and plateau lakes, spanning a significant elevational gradient from the Panzhihua confluence up to altitudes between 3,000 and 4,000 m [3]. These two species are morphologically highly similar, with features such as long beards, lower lips, and hypopharyngeal teeth. Therefore, on the basis of their distinct distribution patterns, *P. leptosomus* and *P. kaznakovi* are likely excellent case studies for investigating the impact of plateau uplift on the speciation processes of fish distributed across different river systems.

This study employed multivariate morphological methods to comparatively analyze the morphological characteristics of the slender leaf catfish and the naked-bellied leaf catfish. Additionally, to analyze the genetic diversity of geographically distinct populations within *P. leptosomus* and *P. kaznakovi* and cover their adaptive characteristics, whole-genome single-nucleotide polymorphism (SNP) markers were screened using genotyping-by-sequencing (GBS) technology. Then, the population structure, population dynamics, and adaptive differentiation were analyzed. By correlating findings with regional geological structures and climate oscillation events, this study addressed the characteristics of adaptive differentiation against the background of plateau uplift. The results provide a solid foundation for the study of the origin of fish diversity in the Shaluli Mountains region.

## Materials and methods

### Sample collection

**Fig. 1:**
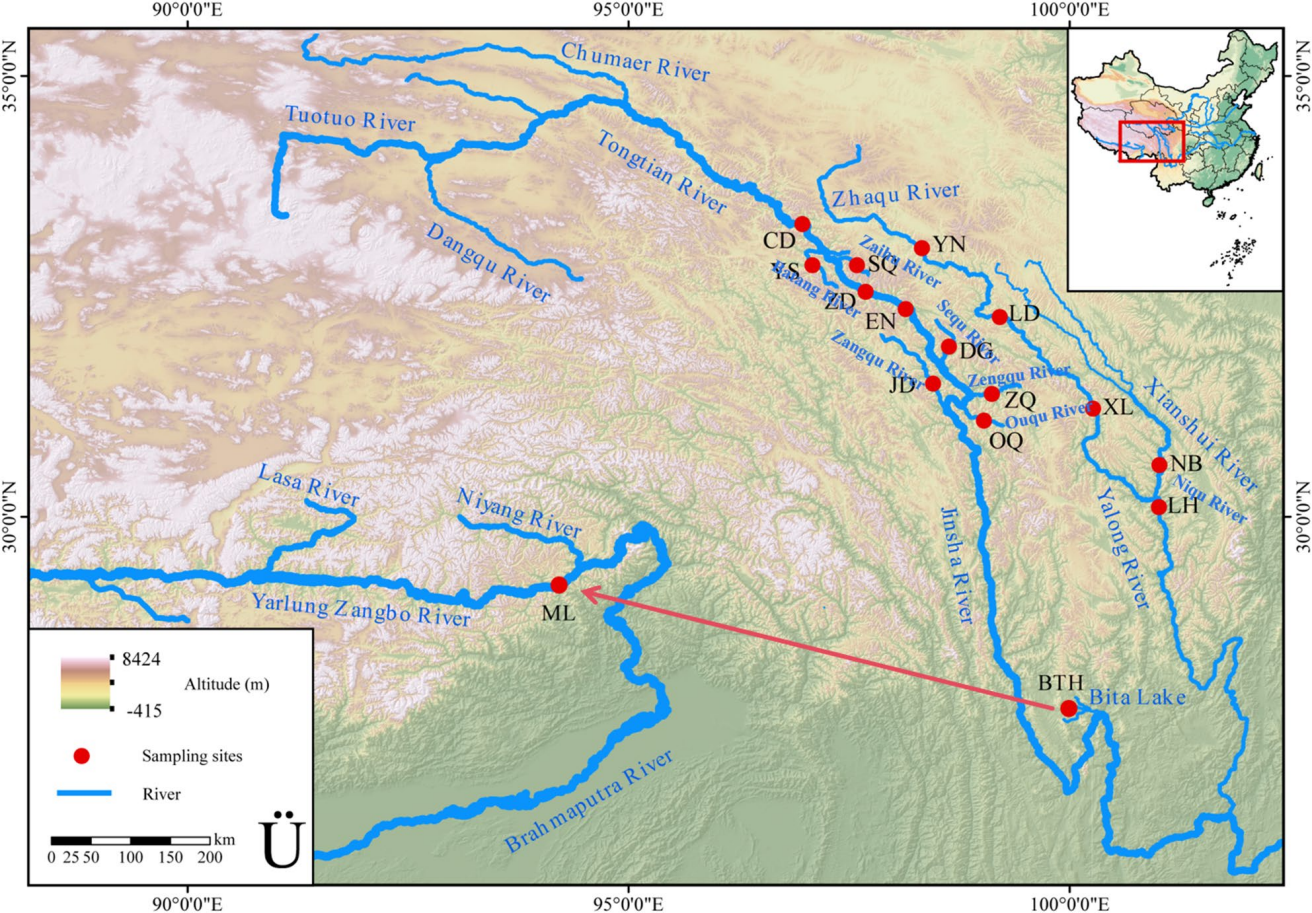
Sampling sites for four *Ptychobarbus species.* N, Yiniu Town; LD, Langduo Town; XL, Xinlong County; LH, Luhuo County; NQ, Ni Qu; CD, Chengduo County; YS, Yushu City; ZD, Zhengda Town; SQ, Shiqu County; EN, Enan Village; DG, Dege County; ZQ, Zang Qu; OQ, Ou Qu; JD, Jiangda County; ML, Milin; Shangri-la, BTH. The red arrow presented the direction of gene flow from BTH to ML

From 2022 to 2024, 84 samples of *P. kaznakovi* were collected from nine sampling sites at the main stream and tributaries of the Tongtian River and Jinsha River, including the Chengduo County section (CD), Zhengda section (ZD), and Enan village section (EN) and the tributaries of the Batang River Yushu section (YS), Zaihuqu Shiqu section (SQ), Sequ Dege section (DG), Zenggqu section (ZQ), Ouqu section (OQ) and Jiangda section (JD) (Fig. 1). Fifty samples of *P. leptosomus* were collected from five sampling sites in the main stream and tributaries of the Yalong River, including the Yiniu section (YN), Langduo section (LD), Xinlong section (XL), Xiuluohai hydropower station reservoir section (LH) and Niqu kanang village section (NQ), which are tributaries of the Xianshui River. A total of 10 fish samples were collected from the Milin section of the middle reaches of the Yarlung Zangbo River. The collected fish were quickly anesthetized with 0.02% tricaine buffer (80 µg/L) (Sigma, SaintLouis, MO, USA). After morphological measurements were performed, pectoral fin samples (1 cm × 1 cm) were cut and immediately preserved in absolute ethanol, followed by storage at − 20 °C for subsequent DNA extraction. The phenol chloroform extraction method [8] was used to extract genomic DNA. The samples were systematically numbered and stored in the endemic fish herbarium of Sichuan Agricultural University in the upper reaches of the Yangtze River. In addition, a total of 3 fin samples of *P. chungtienensis* were provided by the Kunming Institute of Zoology, China, and were collected from Bitahai, Shangri La, Yunnan Province (Table 1). All experimental protocols and investigations were reviewed and approved by the Animal Research and Ethics Committee of Sichuan Agricultural University (Approval No. 20220479) and were conducted in strict compliance with the committee’s established guidelines.

**Table 1:** Data for all samples

Species	Rivers	Sample size/sampling site	Longitude and latitude	Altitude (m)
*Ptychobarbus* *kaznakovi*	Tongtian River	10/Chengduo County(CD)	97.16493056 E 33.08931111 N	3616
	Batang River	10/Yushu City(YS)	97.15174106 E 33.00119815 N	3602
	Jinsha River	10/Zhengda Town(ZD)	97.55528894 E 32.516666 N	3338
	Zaihu Qu	10/Shiqu County(SQ)	97.71396784 E 32.53220952 N	3945
	Jinsha River	10/Enan Village(EN)	98.26735628 E 32.19837932 N	3164
	Se Qu	9/Dege County(DG)	98.57926111 E 31.69600278 N	3105
	Zeng Qu	9/Baiyu County(ZQ)	99.0451442 E 31.39178508 N	3055
	Ou Qu	10/Baiyu County(OQ)	98.84391388 E 31.17272867 N	3035
	Zang Qu	6/Jiangda County(JD)	98.58831667 E 31.22833132 N	2956
*P.* *lepteptosomus*	Yalong River	10/Yiniu Town(YN)	98.307810E 33.062918 N	3906
	Yalong River	10/Langduo Town(LD)	99.051021E 32.263964 N	3635
	Yalong River	10/Xinlong County(XL)	100.271447E 31.345688 N	3011
	Xianshui River	10/Luhuo County(LH)	100.7285847E 31.34568754 N	3150
	Ni Qu	10/Niba village(NQ)	100.2381621E 31.6762026 N	3210
*P.* *dipogon*	Yarlung Zangbo River	10/Mili City(ML)	94.211372E 29.221844 N	2976
*P. chungtienensis*	Bitai Hai	3/Shangri-la(BTH)	99.99148964E 27.82047069 N	3538

*E* east, *N* north

### Morphological analysis

In this study, a combined method of framework-based morphometric distance and traditional morphological indexes was employed to compare and analyze the morphological characteristics of *P. kaznakovi* (*n* = 45) and *P. leptosomus* (*n* = 45) systematically (Table S1 and Figure S1). The framework data were measured using Digimizer software with a precision of 0.01 mm.

The countable traits included the pattern of pharyngeal teeth, gill rakers on the first gill arch, pectoral fins, pelvic fins, branched rays of dorsal and anal fins, lateral line scales, scales above and below the lateral line, and the number of intestinal folds. For measurable traits, individual indicators that could not be measured due to sample issues were treated as missing values. Prior to analysis, data preprocessing was conducted. First, all the morphological data were log-transformed to conform to a normal distribution. The transformed data were subsequently tested for normality using independent sample t tests. Afterward, cluster analysis was performed on the data. To avoid interference from differences in body size among groups in the morphological analysis results, all framework measurement data, except for standard length, were normalized by dividing them by the standard length. To distinguish morphological variations among different groups more effectively, principal component analysis (PCA) was conducted using Origin 24.0 software on the basis of standardized multivariate morphometric data for *P. kaznakovi* and *P. leptosomus.*

### Library construction

After the quality of extracted sample DNA was verified, super GBS sequencing technology was used to construct the sequencing library [9]. In summary, PstI HF/MspI was used for DNA digestion, and T4 ligase was utilized at both ends of the digested fragment to add connectors and barcodes. Then, 300–700 bp fragments were recovered by adjusting the volume ratio of magnetic bead solution to the linked product. The recovered fragments were amplified by PCR with high-fidelity enzymes. Furthermore, the concentration of the PCR product was determined using a Qubit fluorometer and was required to exceed 5 ng/µL. The mixed library was sequenced on an Illumina Nova instrument (PE150).

### Sequencing and SNP calling

High-throughput sequencing was performed using the Illumina HiSeq PE150 sequencing platform. Stack software was used to split the offline data according to barcode and enzyme digestion site information to obtain raw reads for each sample [10]. Fastp software further filtered the quality of the raw reads and obtained the clean reads [11]. BWA software was used to compare the quality-controlled clean reads with the reference genome to assess the similarity between the sample and the reference genome [12]. The depth information of sample sequencing was obtained, and the coverage of sequencing data relative to the reference genome was calculated. The reference genome and its annotation information were provided by Xiao et al. [13]. The insert size distribution of each sample was qualitatively determined [14]. In accordance with the comparison results between the sample genome and the reference genome, the haplotypecaller module in Gatk4 software [15, 16] was used for single-nucleotide polymorphism (SNP, only high-quality biallelic SNPs) and insertion/deletion (indel) detection. VCFtools [16] was used to perform further filtering as follows: (1) the sites with a support depth of no less than 4 were retained; (2) the loci with a minimum allele frequency (MAF) lower than 0.01 were deleted; and (3) at least 80% of the samples could be successfully typed.

### Analysis of population genetic diversity

The relevant indicators of genetic diversity [16], including polymorphism information content (*PIC*), observed heterozygosity (*H*_*o*_), expected heterozygosity (*H*_*e*_) and nucleotide diversity (*P*_*i*_), were calculated using VCFtools. The genetic differentiation index (*F*_*st*_) and genetic distance (*DR*) between different populations were calculated using Arlequin 3.5.1.3 [17].

### Analysis of population genetic structure

The phylogenetic tree was constructed using the neighbor‒joining (NJ) method [18]. The distance matrix was calculated using Treebest software [19]. To evaluate the reliability of the evolutionary tree, and the number of repetitions was set to 1000 [20]. PCA was performed on the obtained SNP markers using Plink2 software [21] to extract the first two feature vectors with the greatest impact. The analysis of population structure was performed using Admixture v1.3.0 software [22]. The range of the K value was set to 1‒10. The optimal number of clusters was determined by minimizing the cross-validation error (CV error).

### Analysis of population historical dynamics and gene flow

The smc + + v0.6.5 [23] software was used to analyze the population dynamic history, which can be used to calculate the change trend of the effective population size (*Ne*) in the time dimension. The generation time was 6 [24], and the mutation rate was 4e-9. The software TreeMix v1.12 [25] was used to analyze population gene exchange.

### Analysis of genome‒environment associations and selective sweep regions

The 19 climate data sets were downloaded from WorldClim (https://www.worldclim.org/data/world clim21.html), and the Spearman correlation coefficient was subsequently calculated among those factors. If the correlation coefficient between two variables was greater than 0.8, one of them was removed [26]. This study used redundancy analysis (RDA) for environmental correlation analysis, which was executed using the *rda* function in the VEGAN software package (version 2.5) [14, 26, 27].

The *F*_*st*_ and *P*_*i*_ values between populations were used for selective elimination analysis [28, 29]. VCFtools software [16] slid the interval (the parameter is– maf 0.05 --max-missing 0.8 --min-alleles 2 --max-alleles 2 --window- *P*_*i*_ 10000 --window- *P*_*i*_ -step 5000). The *P*_*i*_ values of the two species were determined. VCFtools software [16] slid the interval (the parameter is– maf 0.05 --max-missing 0.8 --min-alleles 2 --max-alleles 2 -- *F*_*st*_ t-window-size 10000 -- *F*_*st*_ -window-step 500). The *F*_*st*_ values between the two populations were determined. Finally, the common interval of *P*_*i*_ and *F*_*st*_ was integrated.

For functional enrichment analysis, all selected sites were mapped to terms in the GO (Gene Ontology) and KEGG (Kyoto Encyclopedia of Genes and Genomes) databases [30, 31]. By setting *p* < 0.05 as the significance threshold, the significantly enriched GO terms and KEGG pathways were identified.

## Results

### Analysis of the main external characteristics

**Fig. 2:**
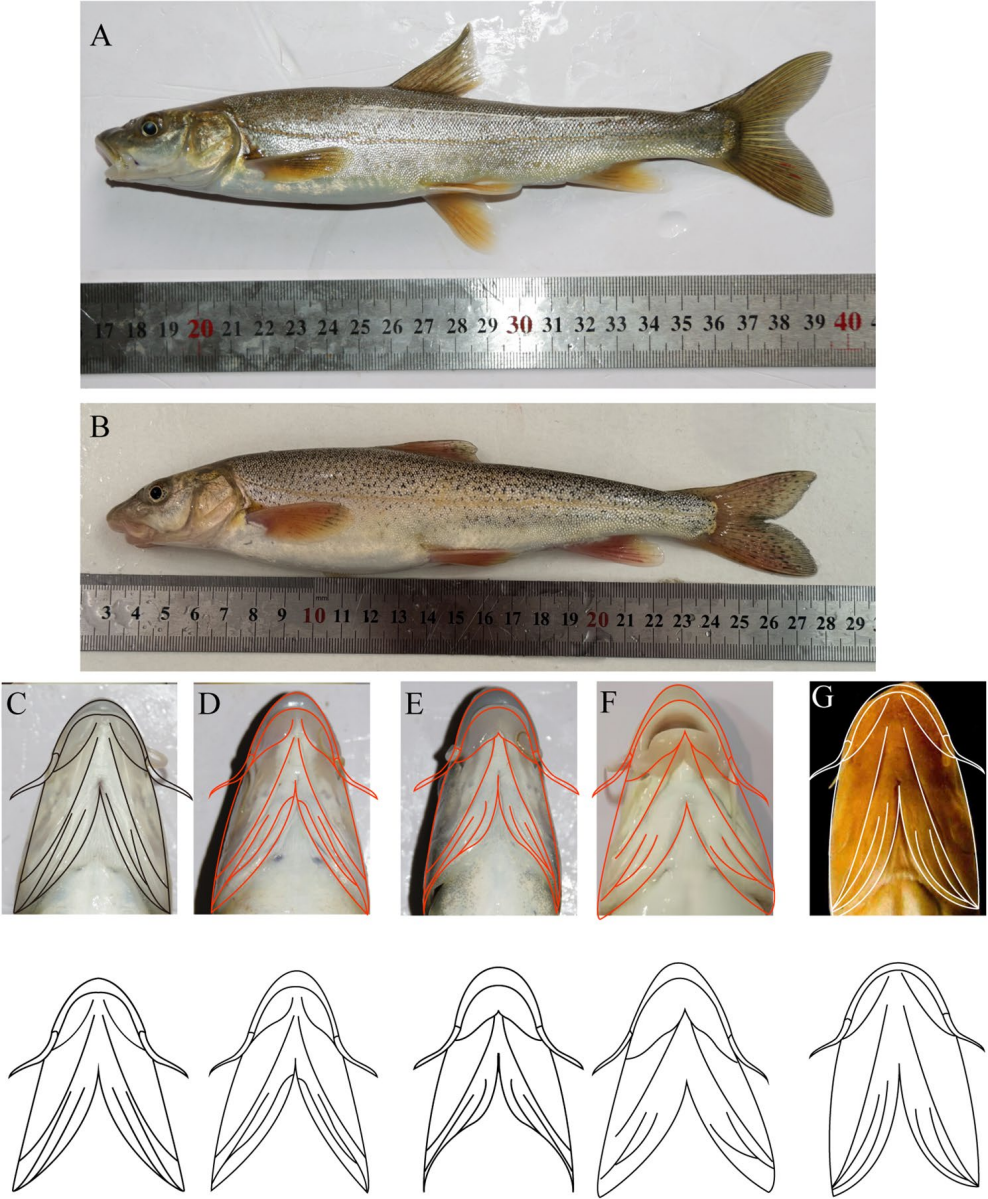
External morphological characteristics of *Ptychobarbus leptosomus* (**A**) and* P. kaznakovi* (**B**) and the lower lip features of *P. leptosomus*, *P. kaznakovi*, and *P. chungtienensis*. Three types of lower lip features in *P. leptosomus* (**C**, **D**, **E**); lower lip features in *P. kaznakovi*. (**F**); and lower lip features in *P. chungtienensis* from Jinsha River Basin (**G**) [6]. The lowercase letters in the fish mouth pictures correspond to uppercase letters in the figure legend

*P. leptosomus* has a bluish-gray color on its sides and back, with fewer spots that are mostly concentrated on the caudal fin, and the ventral side is silvery white (Fig. 2A). *P. kaznakovi* has a dark brownish-black color on its sides and back, with dense and irregular small spots distributed across the body; its ventral side is also silvery white (Fig. 2B). The fins are grayish yellow, with numerous spots on the dorsal and caudal fins, whereas the other fins have fewer spots.

Comparative observation of *P. kaznakovi* and *P. leptosomus* revealed that the lower lip structure of *P. leptosomus* has three characteristics: the left and right lobes are not in contact, there is a small middle lobe, and the left and right lobes are thick and not in contact with each other (Fig. 2C, D and E). *P. kaznakovi* subsequently developed, and the lower lip divided into two lobes, which are in contact at the front (Fig. 2F). This type of lower lip feature was observed in *P. chungtienensis* from the Jinsha River Basin (Fig. 2G) [6]. However, there was no difference in body length or sex (male or female) among all the samples observed.

### Analysis of countable traits

The numbers of gill rakes outside the first gill arch of *P. kaznakovi* and *P. leptosomus* are 11 ~ 15 and 14 ~ 17, respectively. There were ranges overlap in the number of gill rakes outside the first gill arch between *P. kaznakovi* and *P. leptosomus*. However, there was no significant difference in the number of gill rakes, teeth, dorsal fins, pectoral fins, abdominal fins, anal fin branches, lateral line scales, lateral line upper/lower scales, or intestinal bends on the inner side of the first branchial arch (Table 2).

**Table 2:** Statistics of countable traits of *Ptychobarbus Kaznakovi* and *P. leptosomus*

Countable traits	Ptychobarbus kaznakovi	*P*. leptosomus
Number of gill rakes outside the first branchial arch	11 ~ 15	14 ~ 17
Number of gill rakes inside the first gill arch	16 ~ 19	16 ~ 20
Toothed type	2 lines, 3.4–4.3	2 lines, 3.4–4.3
Dorsal fin branching fin	8	8
Branching fin of pectoral fin	17 ~ 19	17 ~ 19
Ventral fin branching fin	8 ~ 10	8 ~ 9
Breech fin branching fin	5	5
Number of lateral scales	94–122	91–122
Number of scales on side line	18–26	20–30
Number of scales under side line	14–20	16–23
Number of bowels	2	2

### Cluster analysis of the morphological indexes

#### PCA of traditional quantifiable traits

**Fig. 3:**
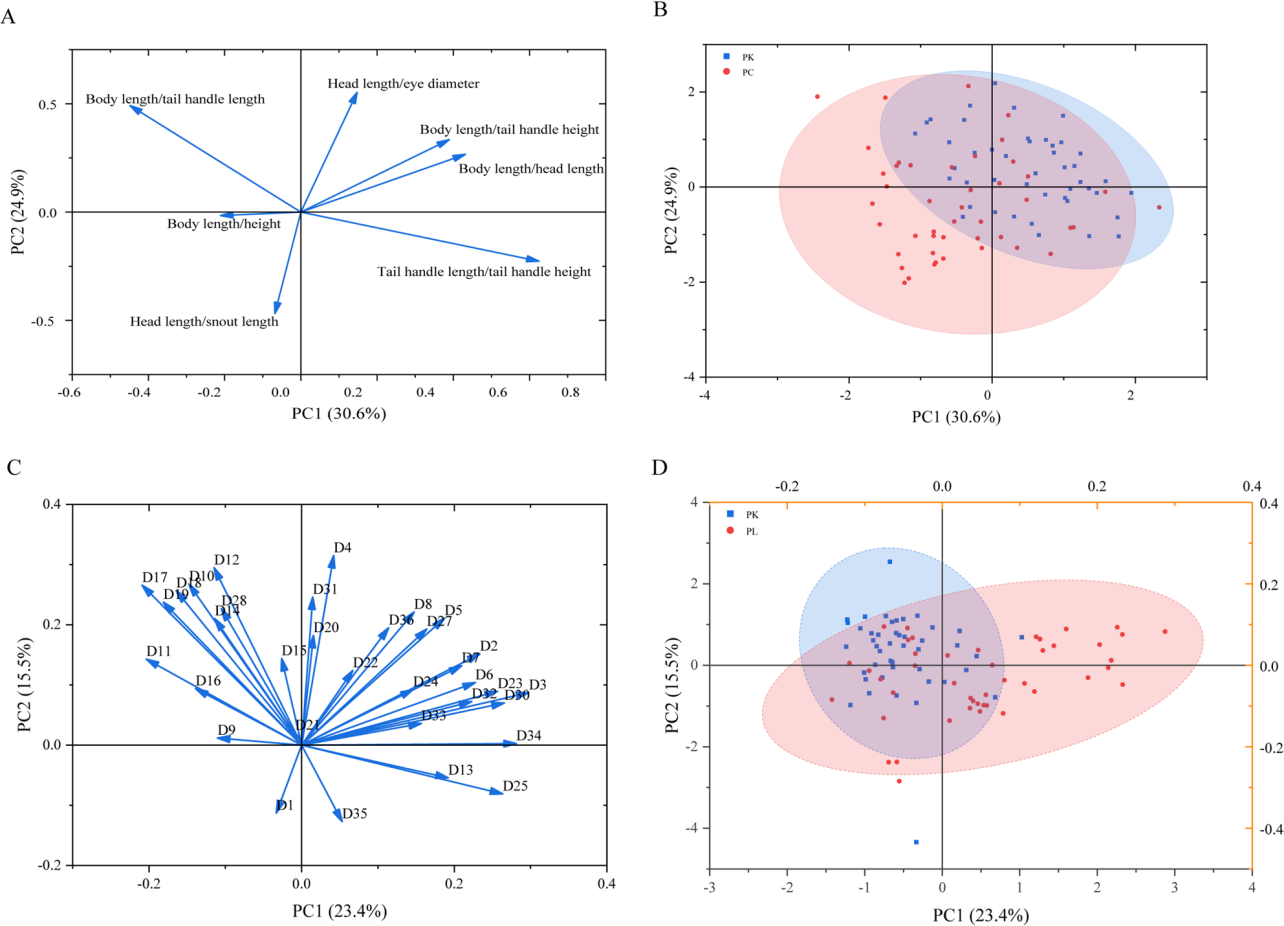
PCA of traditional morphological data and multivariate morphological data of *Ptychobarbus kaznakovi* and *P. leptosomus*. **A**, principal component loading diagram of traditional morphological data; **B**, distribution map of the scattered points of the main components of traditional morphological data; **C**, principal component loading diagram of multivariate morphological data; **D**, distribution map of the scattered points of the main components of multivariate morphological data. PK, *P. kaznakovi;* PL, *P. leptosomus*; PC, principal component

The measurable traits are presented in Table S2. *P. leptosomus* has an elongated body, with a body length that is 4.01‒5.70 times the body height and 6.13‒7.74 times the caudal peduncle length. In contrast, *P. kaznakovi* has a body length that is 4.34‒5.53 times the body height and 5.98‒7.62 times the caudal peduncle length, which is consistent with the morphological description of *P. leptosomus* by a previous study [6]. PCA was used to analyze the proportional traits of *P. kaznakovi* and *P. leptosomus*. Four principal components were obtained, and the results of the variable contribution rates indicate that when the number of extracted principal components reaches four, the eigenvalues begin to show no significant changes. Therefore, four main principal components of the proportional trait data were identified, with eigenvalues of 2.144, 1.740, 1.215, and 0.735. The cumulative contribution rate of the four principal components was 83.37%. The principal component loading plot (Fig. 3A) and the scatter distribution plot (Fig. 3B) show that PC1 and PC2 together explain 55.5% of the total variation (PC1 = 30.6%, PC2 = 24.9%). PC1 is driven mainly by the proportional traits of the tail and head; positive indicators such as body length/caudal peduncle length and head length/orbital diameter reveal the slender tail characteristics of *P. leptosomus*. PC2 is centered on body length/body height, indicating the elongated body shape of *P. leptosomus*. The results suggest that the two species, *P. kaznakovi* and *P. leptosomus*, do not significantly differ in measurable traits. Most individuals are relatively concentrated with overlapping traits. The distribution of traits in *P. kaznakovi* is relatively more concentrated.

#### PCA of multivariate morphological data

After PCA was conducted using the multivariate morphological data of *P. kaznakovi* and *P. leptosomus*, a total of 34 principal components were obtained. An analysis of the variable contribution rates and the scree plot revealed that the eigenvalues did not significantly change after the ninth principal component was extracted. Therefore, there were nine main principal components for the morphometric traits for both species, with eigenvalues of 7.944, 5.258, 3.350, 2.707, 2.025, 1.831, 1.485, 1.176, and 1.017. The cumulative contribution rate of these nine principal components was 87.652%.

The principal component loading plot (Fig. 3C) and scatter plot (Fig. 3D) based on the multivariate morphological data revealed that in the first principal component, indicators such as the distance from the snout tip to the pectoral fin base (D3) and the distance from the post-occipital region to the pelvic fin base (D10) had higher loadings, mainly reflecting the characteristics of the trunk. In the second principal component, indicators such as the length of the caudal peduncle (D35) and the height of the caudal peduncle (D36) had higher loadings, mainly reflecting the characteristics of the tail. *P. kaznakovi* and *P. leptosomus* presented a partially overlapping distribution pattern.

### Sequencing quality

A total of 63 *Ptychobarbus* samples were sequenced by GBS, yielding 424.68 Gb of raw data. After filtering, 384.52 Gb of clean data were obtained. The sequencing quality was high, with a Q20 > 96.50% and a Q30 > 90.89%. A total of 9,695,559 raw SNPs were called, and 2,070,747 high-quality SNPs were detected after filtering.

### Phylogenetic analysis and population genetic diversity

**Fig. 4:**
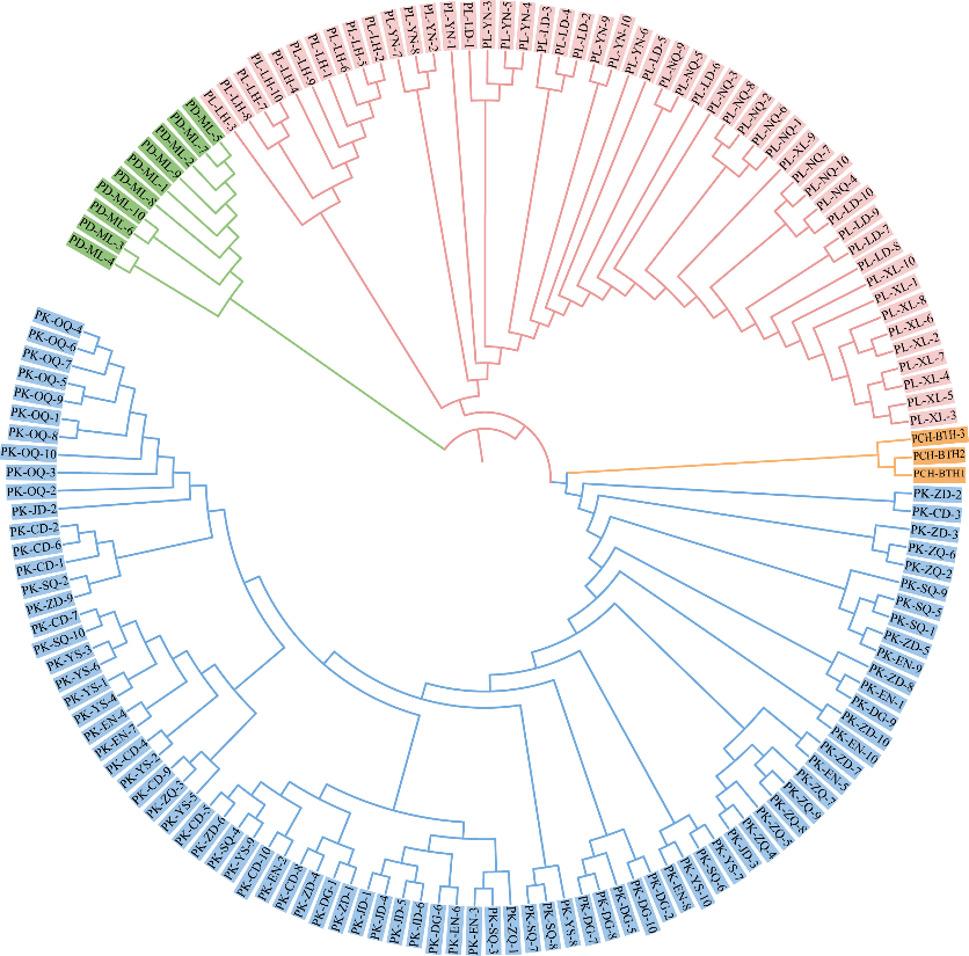
Neighbor-joining evolutionary tree of 147 individuals of *Ptychobarbus leptosomus*,* P. kaznakovi*, *P. chungtienensis*, and *P. dipogon*. PL, *Ptychobarbus leptosomus*, pink color; PK, *P. kaznakovi*, blue color; PCH, *P. chungtienensis*, orange color; PD, *P. dipogon*, green color. YN, Yiniu Town; LD, Langduo Town; XL, Xinlong County; LH, Luhuo County; NQ, Ni Qu. CD, Chengduo County; YS, Yushu City; ZD, Zhengda Town; SQ, Shiqu County; EN, Enan Village; DG, Dege County; ZQ, Zang Qu; OQ, Ou Qu; JD, Jiangda County; ML, Milin

The NJ phylogenetic tree was constructed on the basis of the simplified genome data of *P. dipogon*, *P. leptosomus* and *P. chungtienensis* combined with the resequencing data of *P. kaznakovi* obtained from our research team (Fig. 4). The results revealed that 147 samples could be divided into three branches, and *P. dipogon* were located at the outermost side of the phylogenetic tree. *P. kaznakovi* and *P. chungtienensis* formed a sister group and clustered into one branch, whereas *P. leptosomus* clustered separately.

The genetic diversity index (Table 3) was calculated according to the simplified genomic SNPs. The *H*_*o*_, *H*_*e*_, *P*_*i*_ and *PIC* of *P. leptosomus* were greater than those of *P. kaznakovi*, *P. chungtienensis* and *P. dipogon*. The results revealed that the diversity of *P. leptosomus* was relatively high; additionally, the genetic information was relatively abundant. The observed heterozygosity (*H*_*o*_) was greater than the expected heterozygosity (*H*_*e*_).

**Table 3:** Genetic diversity parameters among *Ptychobarbus leptosomus*,* P. kaznakovi*,* P. chungtienensis* and *P. dipogon*

Population	H_o_	H_e_	Pi	PIC
PL	0.4318	0.2870	0.2883	0.2428
PK	0.4049	0.2734	0.2830	0.2254
PCH	0.2331	0.1446	0.1802	0.1128
PD	0.2812	0.1874	0.2006	0.1477

*H*_*e*_, expected heterozygosity; *H*_*o*_, observed heterozygosity; *PIC* polymorphism information content, *Pi* nucleotide diversity, PL *P. leptosomus,* PK *P. kaznakovi,* PCH *P. chungtienensis,* PD *P. dipogon*

#### Population diversity and genetic divergence

**Fig. 5:**
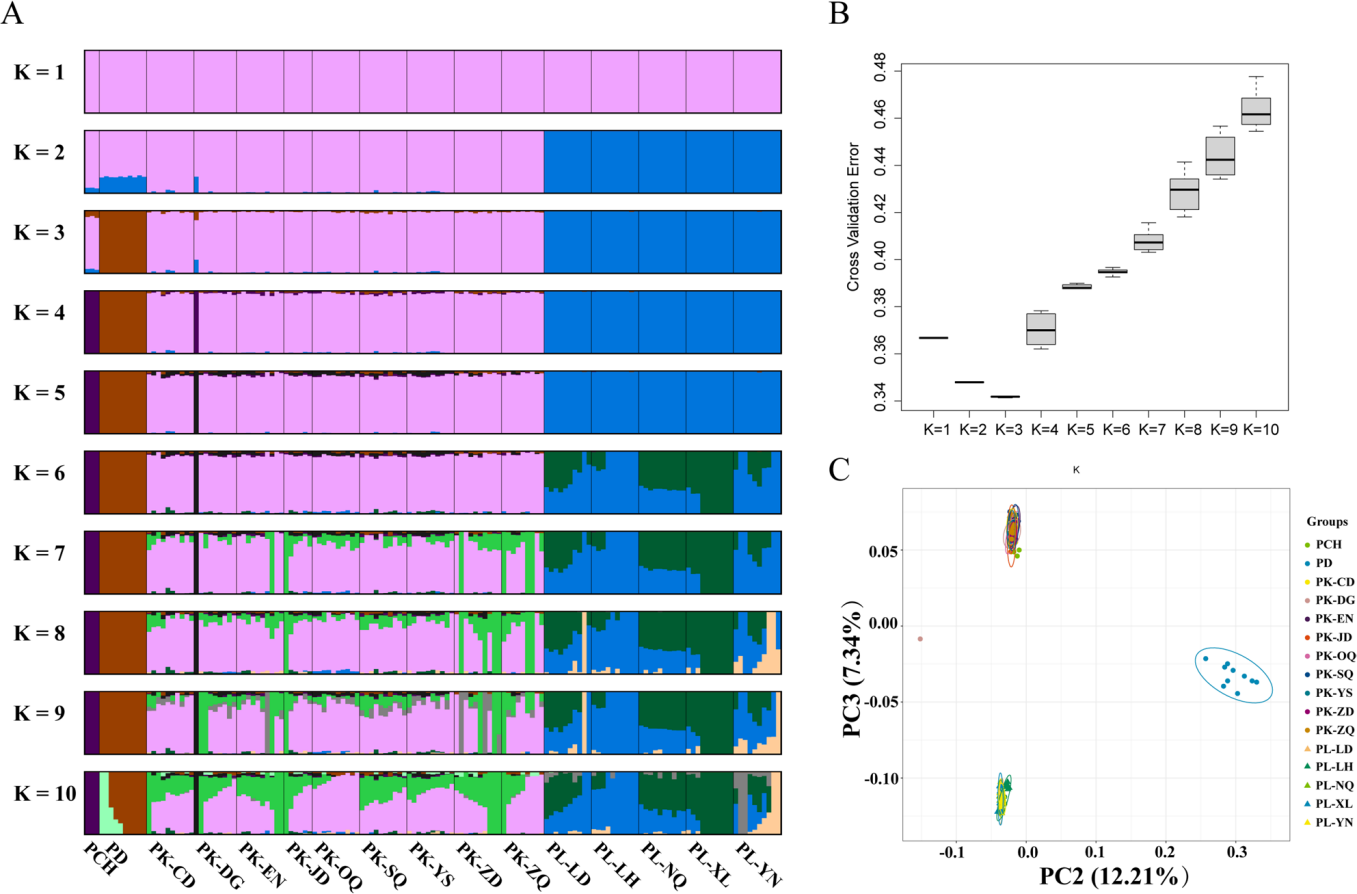
Population structure analysis for *Ptychobarbus leptosomus*, *P. kaznakovi*, *P. chungtienensis*, and *P. dipogon*. **A**, population structure from K = 1 to K = 10; **B**, error rates for the K values of the four *Ptychobarbus* fish admixtures by cross-validation; **C**, principal component analysis for the four *Ptychobarbus* fish. PL, *P. leptosomus*; PK, *P. kaznakovi*; PCH, *P. chungtienensis*; PD, *P. dipogon*. YN, Yiniu Town; LD, Langduo Town; XL, Xinlong County; LH, Luhuo County; NQ, Ni Qu. CD, Chengduo County; YS, Yushu City; ZD, Zhengda Town; SQ, Shiqu County; EN, Enan Village; DG, Dege County; ZQ, Zang Qu; OQ, Ou Qu; JD, Jiangda County; ML, Milin. PC, principal component

On the basis of the analysis of population genetic structure, we observed the distribution of genetic structure under different K values (the K value represents the number of assumed ancestors) (Fig. 5A and B). The results showed that when K = 3, the analysis results were the most suitable as the basis for grouping. The four species of *Ptychobarbus* were divided into three groups at the level of population genetic structure analysis, which indicates that *P. kaznakovi* and *P. chungtienensis* may have originated from the same ancestral group. With increasing K content, *P. leptosomus* differentiated earlier and formed independent genetic components, indicating that there were significant genetic differences between the geographical populations of *P. kaznakovi* and *P. leptosomus*.

To further analyze the population genetic structure characteristics of the two species, we used PCA to reveal the relationships among those populations. The PCA results revealed that PC2 explained 12.21% of the variation and PC3 explained 7.34% of the variation (Fig. 5C). This result suggests that the samples of different species of *Ptychobarbus* present certain distribution characteristics in the principal component space composed of PC2 and PC3. Cluster analysis of the *P. kaznakovi* and *P. chungtienensis* samples and the *P. dipogon* and *P. leptosomus* samples presented geographically nonoverlapping ranges. Phylogenetic analyses confirmed that *P. leptosomus*, *P. kaznakov*i, *P. chungtienensis*, and *P. dipogon* constitute distinct species clusters.

#### Historical population dynamics of *P. leptosomus* and *P. kaznakovi*

**Fig. 6:**
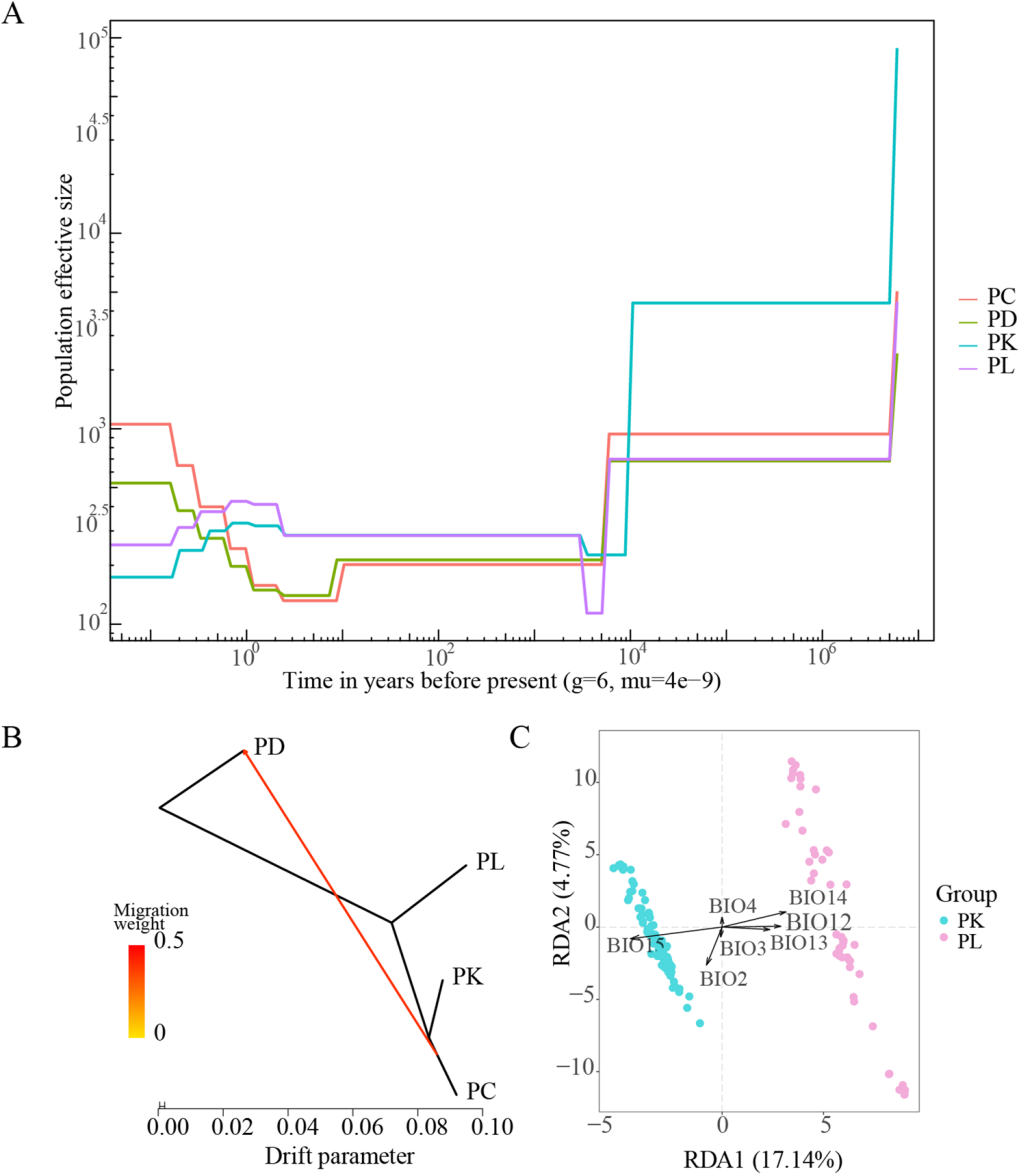
Analysis of historical population dynamics, gene flow, and environmental adaptability and genetic variation of four species in *Ptychobarbus*. A, estimation of historical effective population size; B, flow analysis; C, RDA of genetic variation and environmental variables. PL, *P. leptosomus*; PK, *P.* *kaznakovi*; PC,* P. chungtienensis*; PD, *P. dipogon*. Bio2, diurnal range; Bio3, isothermality; Bio12, annual precipitation; Bio13, precipitation of wettest month; Bio14, precipitation of driest month; Bio15, precipitation seasonality and altitude.

According to the analysis of historical population dynamics (Fig. 6A), the populations of *P. leptosomus* and *P. kaznakov*i experienced relatively similar population contractions and expansions. At approximately 10.0 Ma, the Ne values of the two fish species sharply decreased. The effective population sizes of four species subsequently remained relatively stable over approximately 6.0–1.0 Ma. However, at approximately 0.8–0.2 Ma, the effective population sizes of the PL and PK decreased again and then stabilized at approximately 1.5–0.5 Ka. Otherwise, the population sizes of PC and PD experienced a brief decline between 0.1 and 1 Ma, but began to increase after 1 Ma, and stabilized around 0.5 Ka. This dramatic change in the effective size of the recent population may imply that some recent ecological or environmental factors significantly impacted these two species.

#### Gene flow

To clarify the direction of gene flow among the four species, we used TreeMix analysis to construct an ML tree (Fig. 6B). In the presence of gene flow, the four species exhibited lineage differentiation and simulated a migration event on different branches. The gene flow from *P. kaznakovi* and *P. chungtienensis* branches to *P. dipogon* is more obvious. In general, the results of the gene flow analysis were consistent with results from principal component analysis and population genetic structure analysis.

#### Environmental adaptability and genetic variation

Among the whole-genome SNPs distributed across 13 populations of *P. leptosomus* and *P. kaznakovi*, 35,654 were associated with the environmental factors Bio2 (average daily range), Bio3 (isothermal), Bio4 (seasonal temperature), Bio12 (annual precipitation), Bio13 (precipitation in the wettest month), Bio14 (precipitation in the driest month) and Bio15 (seasonal precipitation). The variance interpretation rates of the first two factors, rda1 and rda2, in the RDA were 17.14% and 4.77%, respectively, and Bio15 contributed the most to the genetic variation of the two species (Fig. 6C). The arrow of the Bio14 environmental factors pointed roughly from left to right, which also had a significant impact on the distribution of species and might be one of the important factors leading to the distribution difference between the two species in the horizontal direction.

#### Genes experiencing selective sweeps in *P. leptosomus* and *P. kaznakovi*

**Fig. 7:**
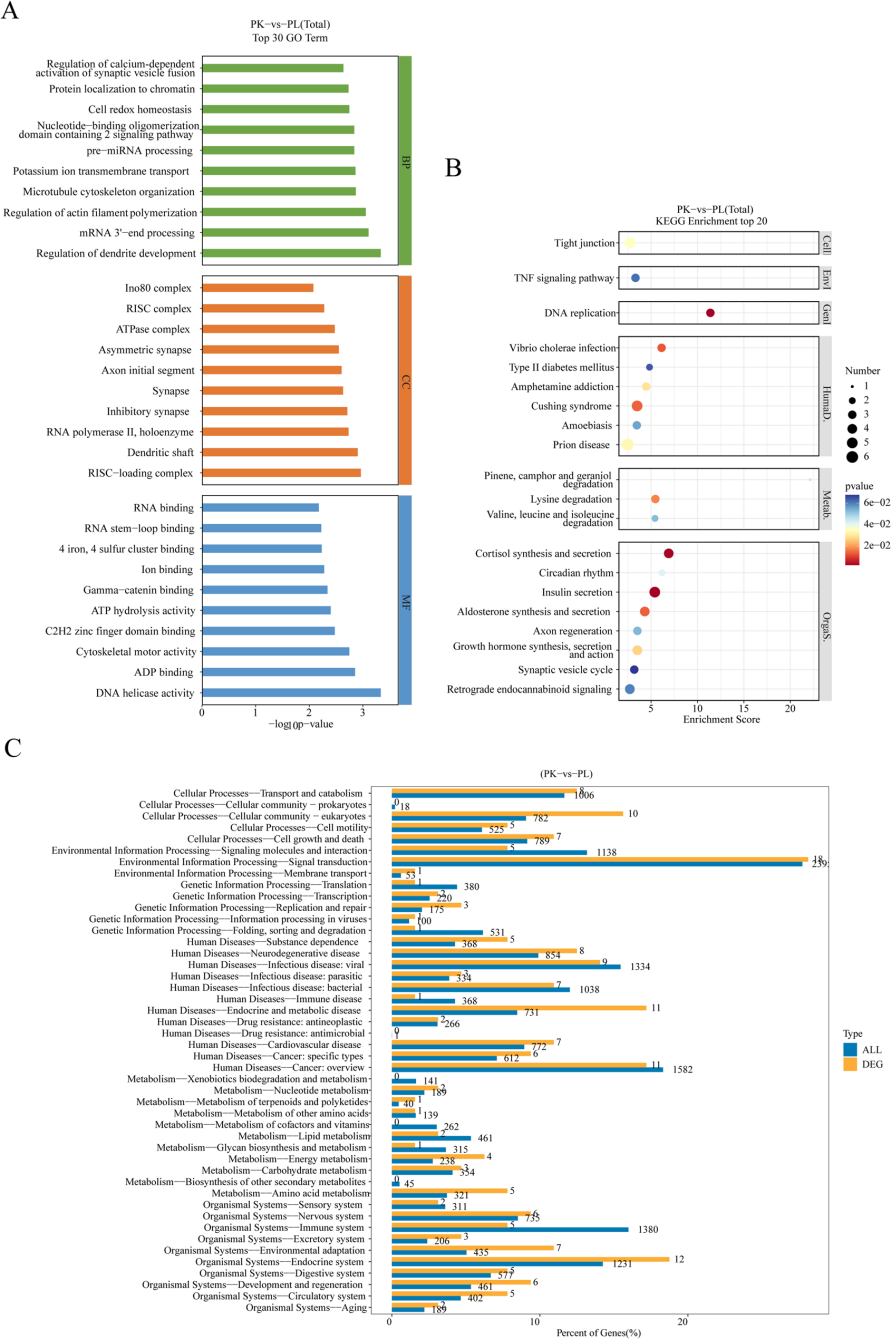
GO and KEGG enrichment analyses of genes experiencing selective sweeps. **A**, the top 30 GO pathways significantly enriched in *Ptychobarbus kaznakovi* and *P. leptosomus*; **B**, the top 30 KEGG pathways significantly enriched; **C**, comparative analysis of KEGG level 2 pathway distributions between differentially expressed genes

The *P*_*i*_ ratio and *F*_*st*_ information of the two populations were integrated. A window with a small or large *P*_*i*_ ratio and a large *F*_*st*_ value as the selection clearing area (the threshold is set to 5% this time) was selected and annotated. Through comparative analysis of *P. leptosomus* and *P. kaznakovi*, the selective sweep regions were identified, and GO and KEGG enrichment analyses were conducted for the candidate genes on the basis of SNP sites in these regions. The clearly selected regions shared by the two species were defined by pipk/pipl ≥ 2.43 and *F*_*st*_ ≥ 0.38. GO enrichment analysis revealed that the selected genes were annotated with 227 terms, 139, 38 and 50 of which belonged to the biological process, cellular component and molecular function subgroups, respectively. The biological process category was related mainly to protein localization to chromatin, cell redox homeostasis and pre-miRNA processing. The GO enrichment of the cell component categories was related mainly to the INO80 complex and ATPase complex. The enriched molecular functions were related mainly to ADP binding, ATP hydrolase activity, DNA helicase activity and other energy metabolism pathways (Fig. 7A). KEGG enrichment analysis (Fig. 7B and C) revealed that these genes (such as *gldc*, *glycine decarboxylase*; *cyp51*,* igf-1*, and *tnf-α*) were involved mainly in glycine, serine and threonine metabolism; glycan biosynthesis and metabolism; growth hormone synthesis, secretion and action; the TNF signaling pathway; and other related pathways.

## Discussion

### Cluster analysis of the morphological indexes

For many years, the classification of *Ptychobarbus* fish has been a contentious issue among taxonomists, especially regarding the species *P. leptosomus*, *P. kaznakovi* and *P. chungtienensis*. Zhang et al. described *P. leptosomus* on the basis of a single dissected specimen and noted that it is morphologically very similar to *P. chungtienensis*. However, they argued that it is a distinct and valid species separate from both *P. kaznakovi* and *P. chungtienensis* and identified it as a new species [6]. In 2020, Guo et al., in the “Atlas of Fishes of Sichuan”, suggested that *P. leptosomus* should be considered a synonym for *P. chungtienensis* [32].

This study compared the body color and lower lip characteristics of *P. leptosomus* and *P. kaznakovi*. The lower lip of *P. kaznakovi* is well developed and is divided into two lobes, with connection between the anterior parts of the two lateral lobes. In contrast, the lower lip of *P. leptosomus* exhibits different structural features, including two separate left and right lobes, a small middle lobe, and the left and right lobes are thick and not in contact with each other. These characteristics are consistent with the description of the lower lip morphology of 11 specimens of *P. chungtienensis* by Guo et al. [112]. Additionally, Chen et al. reported that the lower lip structure of *P. kaznakovi* also includes the above three morphological features. However, after 45 specimens of *P. kaznakovi* were observed in this study, it was found that their lower lip structure did not exhibit those three morphological features. Moreover, the analysis of countable traits revealed differences in the number of gill rakers on the outer side of the first gill arch between *P. kaznakovi* (11‒15) and *P. leptosomus* (14‒17). This differs from the results obtained by Guo et al., who reported that the number of gill rakers on the outer side of the first gill arch of *P. chungtienensis* was 13‒14 [32]. In summary, by comparing *P. kaznakovi* and *P. leptosomus*, this study revealed significant morphological differences between the two species and highlighted the similarities and differences compared with previous research findings, providing important morphological evidence for further distinguishing these two fish species.

The analysis of measurable traits revealed that *P. leptosomus* has an elongated body, with a body length that is 4.01‒5.70 times the body height and 6.13‒7.74 times the caudal peduncle length. *P. kaznakovi* has a body length that is 4.34‒6.53 times the body height and 5.98‒7.62 times the caudal peduncle length. These findings overlap with previous morphological descriptions. For instance, Zhang et al. [6] reported one *P. leptosomus* (body length/height = 5.94; body length/caudal peduncle length = 7.12) and 12 *P. kaznakovi* (4.2‒5.9; 6.0‒7.5). Guo et al. [32] described 11 *P. leptosomus* (4.61‒5.05; 5.68‒7.12) and 12 *P. kaznakovi* (4.90‒5.59; 4.85‒5.38), and Chen et al. [33] described 15 *P. kaznakovi* (4.30‒5.50; 6.00‒7.30). Ding et al. [34] described two samples of *P. kaznakovi* (5.20–5.30; 6.10–6.20), and Wu et al. [7] described 75 *P. kaznakovi* (4.39‒6.88; 5.16‒6.89) and 20 *P. chungtienensis* (body length/height = 4.42‒7.35; body length/caudal peduncle length = 5.71‒7.35) that were found in only specific locations in Yunnan Province, such as Napahai, Bitahai, and Xiao Zhongdian. These data overlap with previous findings, indicating that the ratios of body length to body height and caudal peduncle length are somewhat similar between the two fish species but also differ. For example, the range of the body length/body height ratio for *P. kaznakovi* across different studies is 4.2‒6.9, and the range of the body length/caudal peduncle length ratio is 5.0‒7.9. For *P. leptosomus*, the range of the body length/body height ratio is 4.6‒7.4, and the range of the body length/caudal peduncle length ratio is 5.7‒7.8. These differences may be related to factors such as sample size, geographical distribution, and individual age. The overlap and minor differences in measurable traits between *P. leptosomus* and *P. kaznakovi* may be closely related to their living environments and ecological habits. The slender body of *P. leptosomus* is suitable for rapid swimming, whereas the more diverse body shape of *P. kaznakovi* may be adapted to a wider range of ecological environments.

The multivariate morphological analysis revealed that the PCA based on 34 framework measurements yielded nine principal components with a cumulative contribution rate of 88.74%, which is higher than that of the traditional measurement traits (83.37%). The principal component results indicated that the samples of *P. leptosomus* and *P. kaznakovi* largely overlapped and could not be completely separated. The reason may be that the *P. leptosomus* and *P. kaznakovi* populations are geographically close, and the environmental and climatic conditions are similar, resulting in similar selective pressures. The morphological characteristics of fish are the result of their evolutionary adaptation to the environment. A typical example is the Mexican cavefish (*Astyanax mexicanus*), which has evolved several features related to cave life in response to food scarcity and the darkness of its environment; these traits include reduced pigmentation, smaller eye size, enlarged nonvisual sensory organs, increased body fatness, and reduced sleep duration [35]. A previous study revealed that the morphological differences between *Xenophysogobio boulengeri* and *X. nudicorpa* are related primarily to eye diameter, fin length, and barbel length [36]. Similarly, the long-finned snailfish (*Rhinogobio ventralis*) shows evolutionary adaptations to different water flow environments through features such as a robust caudal peduncle, smaller eye diameter, and shorter snout [37]. Therefore, the differences in caudal peduncle and fin length between *P. leptosomus* and *P. kaznakovi* are not significant, which may be due to the similar geographical environments they inhabit.

#### Characteristics of species differentiation and gene flow in *P. leptosomus* and *P. kaznakovi*

Orogeny since the Cenozoic Era and its accompanying environmental changes have shaped a broad and complex geomorphic pattern throughout the QTP [38–40]. In the Hengduan Mountains region, high mountains and deep valleys alternate within a very short straight-line distance, making it an “ecological island” isolated from the surrounding lower regions [41, 42]. Within the vast geographical range of the QTP, many endemic species with close relationships are distributed in diverse habitats along altitudinal gradients and with spatial heterogeneity [43, 44]. Therefore, strong geographical isolation has long been considered the main driving force of species differentiation in the region [45–48]. However, due to the extremely high habitat diversity in the region, ecological selection may play an important role by promoting reproductive isolation and potential “neighborhood species formation“ [49].

In this study, the PCA results revealed that *P. kaznakovi*, *P. leptosomus* and *P. dipogon* formed distinct clusters but that *P. chungtienensis* and *P. kaznakovi* clustered together. Through an analysis of genetic structure, it was found that when K = 3, there were three populations at the level of population genetic structure analysis. Among them, *P. chungtienensis* and *P. kaznakovi* were clustered, which was consistent with the PCA results. This phenomenon of population differentiation is influenced mainly by ancient climate fluctuations and changes in river systems. These factors hinder gene flow between populations, resulting in the differentiation of a single population into two or several populations. This phenomenon has also been reported in other freshwater fish [50, 51]. Spatial heterogeneity causes disproportionate selection pressure generated by heterogeneous habitats to become the main driving force of adaptive species formation by weakening gene flow [52, 53]. These findings not only reveal the primary mechanisms of population differentiation in leaf whisker catfish but also provide an important reference for understanding the speciation and evolution of other freshwater fish species.

TreeMix analysis revealed that gene exchange among *P. chungtienensis*, *P. kaznakovi* and *P. leptosomus* was weak. Introgression hybridization between different lineages may play an important role in the differentiation of *Schizothorax* species. Due to large-scale river restructuring and river seizing events triggered by the uplift of the QTP, *Schizothorax* has experienced many complex gene penetration events [54–56]. Located between the Jinsha River and the Yalong River in the southeast of the QTP, the Shaluli Mountains are the largest mountain range in the Hengduan Mountains. With the uplift of the QTP, the altitude continues to rise, which is an important factor affecting the adaptive differentiation of species in the southeastern QTP [3, 4]. The geological barrier between the Jinsha River and Yalong River water systems (the uplift of the Shaluli Mountains) played a key role in differentiating between the populations of *P. kaznakovi* and *P. leptosomus* distributed on both sides of the Shaluli Mountains. This phenomenon has also been reported in *Ligularia tongolensis* [57], *Cyananthus delavayi* [58], *Batrachuperus pinchoni*i [59], *Nivivanter superior* [60], *Tetraophasis obscuru*s and *Schizopygopsis malacanthus* [61]. The above studies indicate that topographic changes in these regions (such as the uplift of the QTP and the Shaluli Mountains) caused intraspecific and interspecific differentiation through allochthonous differentiation [57, 58, 62] and led to fish migration and introgressive hybridization between lineages, which indicates that the fish in these areas have responded to climate oscillations and geological events. Although the distribution range of *P. leptosomus* was limited, the species presented greater nucleotide diversity than *P. kaznakovi*, *P. chungtienensis* or *P. dipogon*. This high nucleotide diversity enables *P. leptosomus* to persist in its narrow distribution range and enhances its adaptability to the environments of the high mountains and valleys of the Shaluli Mountains. In conclusion, the geological activities of the Tibetan Plateau and its surrounding mountain ranges (such as the Shaluli Mountains), including uplift, reorganization of river systems, and river capture, have played crucial roles in the speciation and adaptive evolution of schizothoracine fishes.

#### Historical dynamics of the populations of *P. leptosomus* and *P. kaznakovi*

Plateau fish originated and evolved with the uplift of the plateau, and their distribution in various river systems is the result of the headwater capture of these river systems [63]. Most studies indicate that climate oscillations and geological events are important factors affecting the geographical distribution patterns and genetic differentiation of species on the QTP [64–66]. The climatic oscillations since the late Pliocene are believed to have promoted the speciation of polyploids, including allopolyploids and autopolyploids, in plants from the QTP. Moreover, polyploids are common not only in plants but also in fish on the QTP [67]. The uplift of the QTP facilitated the widespread dispersal and diversification of the diploid ancestors of *Schizothorax* fish. Subsequently, the climatic and environmental changes during the Pliocene–Pleistocene enabled the diploid ancestors of *Schizothorax* fish to adapt through autopolyploidization. With the ongoing climatic and environmental changes in the Pliocene–Pleistocene, polyploid *Schizothorax* fish gradually replaced their diploid ancestors, further driving the diversification of *Schizothorax* fish in the surrounding regions of the QTP [68, 69]. After a long period of evolution, these isolated populations may evolve into new subspecies or even new species.

This study provides an in-depth analysis of the associations between the historical population dynamics of *P. kaznakovi* and *P. leptosomus* and regional geological events. On the basis of the results of historical population dynamics, we observed that the two species experienced similar population contraction and expansion patterns. During the late stage of the second uplift of the QTP (10.0–4.0 Ma), climate change caused by the monsoon cycle led to rapid global cooling [70]. Concurrently, the effective population sizes of *P. kaznakovi* and *P. leptosomus* decreased sharply during this period. The *Ne* values of the two species subsequently remained relatively stable during the period of approximately 5.0–1.0 Ma, indicating that the populations may have experienced a period of adaptation or equilibrium. The Shaluli Mountain region [68] has experienced several periods of significant uplift since the Quaternary, of which the earliest glaciation occurred at approximately 0.6–0.5 Ma. Additionally, the Jinsha River and Yalong River tributaries strongly cut down the middle of the mountain, forming the initial canyon landform [71]. The effective population sizes of PK and PL showed a downward trend again (0.8–0.2 Ma). It is speculated that the historical dynamics of the populations of PK and PL are likely affected by the Shaluli Mountain ice age. The *Ne* values of the two species tended to be stable at approximately 1.5–0.5 Ka. The planation surface formed in the late Tertiary of the Shaluli Mountains disintegrated from the end of the Pliocene to the early Quaternary, and rift basins [4] appeared in some areas of the planation surface, which received fluvial lacustrine deposits. The plateau subsequently experienced continuous pulsating uplift, accompanied by tectonic faulting and the extension and cutting of several major tributaries of the Jinsha River and Yalong River, forming several faulted river valleys and earlier terraces. The above results show that tectonic events in the Shaluli Mountains and Quaternary climate oscillation have important impacts on the current distribution patterns of *P. kaznakovi* and *P. leptosomus* in the study area. Due to the uplift of the Shaluli Mountains, the ancestral population was dispersed between the Jinsha River and the Yalong River, resulting in a lack of connection between the phylogenetic structure and the water system. After this differentiation, the regional population was limited by geographical barriers and the species’ preference for high-altitude environments. The Shaluli Mountain area may have undergone four major glacial events, and experienced a significant uplift after the earliest glacial event (5.71 ka BP). Then, during the early and late periods of MIS3 (approximately 60–37.8 ka BP), the summer monsoon with abundant precipitati and high temperature was strong, and the water and heat balance conditions were conducive to the development of glaciers. In the middle period of MIS3, the temperature was lower, the summer monsoon was stronger, and the precipitation was more abundant. This cold and humid climate condition was favorable for the development of glaciers [72]. As the Quaternary period progressed into its later stages, it was accompanied by the accumulation of loess, especially in the northern Yalong River valley [73]. It can be inferred that the above factors may affect the extent of population contraction between the PK (Jinsha River) and PL (Yalong River) populations in this period. Though PL and PD, PC and PK were clustered in the same branch, respectively. However, PL and PK had the different patterns of historical dynamics after about 0.1 Ma. It may be due to the above factors. Thus, a long-term independent evolutionary history in the subsequent climate oscillation was experienced, and most of the population experienced expansion and bottleneck events during the climate oscillation. A previous study reported that *S. malacanthus* distributed in this flora also experienced similar expansion and bottleneck events [68]. Therefore, the uplift of mountains and the erosion of glaciers may lead to the isolation and fragmentation of habitats and subsequently affect the gene flow and genetic diversity of *P. kaznakovi* and *P. leptosomus* populations.

#### Characteristics of the environmental adaptability of *P. leptosomus* and *P. kaznakovi*

Due to differences in ecological environments, populations may experience selective pressures from specific environmental factors, leading to selective sweeps at key genetic loci, thus driving population differentiation [74, 75]. Subsequently, dominant mutations may be retained through selection, leading to their proliferation and expansion, which in turn continuously promotes genetic differentiation within populations. This is a common phenomenon in closely related species that are distributed adjacently but occupy different ecological niches [76, 77]. Understanding the genetic basis of plateau fish helps us understand their environmental adaptability [78–80].

In this study, selective elimination analysis was used to explore the genomic adaptation characteristics of *P. kaznakovi* and *P. leptosomus*. The results of GO and KEGG enrichment analyses revealed that the genes significantly enriched in genomic regions under selection for the two fish species were related to disease, the immune system and environmental adaptation (*P* < 0.05). The highly enriched GO terms included protein localization on chromosomes, ATPase complex, DNA helicase activity and ATP hydrolase activity, indicating key roles in gene expression regulation, DNA replication and repair, energy metabolism and other processes. These results suggest that the selective sweep of genes in the region may play a key role in the metabolic adaptability and physiological function of *P. kaznakovi* and *P. leptosomus*. In extreme plateau environments, both low temperature and ultraviolet radiation can cause DNA damage [81–85]. DNA repair is essential for maintaining the integrity and stability of the genome, which ensures the accurate transmission of genetic information and the normal function of cells. In studies of other plateau fishes, such as *S. malacanthus* and *S. pylzovi*, GO enrichment analysis revealed that the positively selected genes were related primarily to DNA repair. The positive selection of these genes helps them adapt to high-altitude areas and strong ultraviolet radiation [86]. The selected sites in *S. oconnori* are associated mainly with amino acid metabolism, the Fanconi anemia pathway and other pathways related to DNA repair and energy metabolism [87]. Homologous recombination and p53 signaling pathways were identified in *Trilophysa bleekeri* as forming an integrated DNA repair mechanism to cope with the extreme environment of the plateau [88]. In summary, both selective sweep analysis and functional enrichment revealed multiple key genes related to metabolic adaptation, physiological function, and DNA repair in the genomes of *P. kaznakovi* and *P. leptosomus*. Their adaptive characteristics are consistent with the DNA repair mechanisms of other plateau fish species in response to extreme environments such as low temperature and intense ultraviolet radiation. This research provides important evidence for elucidating the molecular evolutionary mechanisms of environmental adaptation in plateau fish species.

Fish usually adapt to abiotic conditions, such as temperature, through positive selection, which leaves signatures of selective sweeps in genes associated with the selected genes [89]. Plateau fish exhibit significant adaptive evolutionary characteristics in terms of energy metabolism in environments with low water temperatures and large diurnal temperature variations. Study has shown that, compared with the model organism zebrafish (*Danio rerio*), *Gymnodiptychus pachycheilus* evolves at a faster rate, with selected genes significantly enriched in energy metabolism-related pathways [90]. Compared with those of *Ctenopharyngodon idella* and other low-altitude fishes, the dN/dS ratio of *Schizothorax* fish was significantly greater. Additionally, multiple GO terms related to plateau adaptation, such as energy metabolism, also showed significantly increased evolutionary rates. These findings indicate that plateau fish have undergone a rapid evolutionary process in adapting to the plateau environment [91]. For example, *Glyptosternum maculatum* inhabits high altitudes and exhibits more selected genes that are related mainly to the hypoxia response and energy metabolism [92]. The selected genes of *S. oconnori* were enriched in amino acid metabolism, glucose metabolism, lipid metabolism and other pathways [87]. The *Triplophysa* fish presented results similar to those of *Schizothorax*, and the genes with positive selection and rapid evolution signs were also significantly enriched in the energy metabolism category [93]. Therefore, plateau fish species retain adaptive signatures in their genomes through positive selection and selective sweeps, and the selected genes are generally enriched in pathways closely related to environmental adaptation, such as energy metabolism, carbohydrate metabolism, and amino acid metabolism. This study further revealed that the selected genes in *P. kaznakovi* and *P. leptosomus* are enriched in carbohydrate metabolism, growth hormone synthesis and secretion, and the conserved Wnt signaling pathway. These findings suggest that these pathways may be the key molecular mechanisms by which these two species adapt to different aquatic environments on plateaus, providing a new perspective for understanding the ecological adaptive evolution of plateau fish species.

In this study, we found that the selected genes of the two fishes were enriched mainly in amino acid metabolism, glucose metabolism, growth hormone synthesis and secretion. Glucose metabolism is one of the key mechanisms by which the body obtains energy. Its main function is to maintain stable blood glucose concentrations, which are essential for the survival of aquatic organisms such as fish. Through the effective regulation of glucose metabolism, the body can ensure that the blood glucose level is within an appropriate range to provide continuous energy support for the physiological activities of fish and ensure their normal growth and survival [94]. The synthesis and secretion of growth hormone mainly regulate the individual growth rate and energy distribution [95], which may reflect the adaptation of the two species to different niche resource utilization strategies [96]. Mark et al. [96] proposed that fish adjust their energy allocation adaptively during their life cycle and/or respond to changes in environmental conditions through the coordinated regulation of various hormones. In addition, the enriched Wnt signaling pathway is a highly conserved signaling pathway maintained in the process of species adaptive differentiation and plays a key role in the formation of the primary somatic axis, cell differentiation and tissue homeostasis during development [97]. Wnt signaling pathway enrichment has been reported in studies of *S. kozlovi* [98] and *D. rerio* [99]. Therefore, we speculate that the selected genes of the two fishes are enriched in the pathways of glucose metabolism, amino acid metabolism and growth hormone synthesis and secretion to better adapt to the unique water environment of the plateau.

## Conclusion

In summary, the results of this study emphasize the significant role of geological and environmental changes in shaping the population history and evolutionary processes of *P. kaznakovi* and *P. leptosomus* and provide data for understanding the adaptive differentiation and biodiversity of the two species on the QTP. To supplement the study of the effects of geological and environmental changes on the population history and evolutionary process of these two species from the perspective of gene expression, further research can be combined with transcriptomic and other data. Additionally, extensive ecological studies, such as investigations into the habitat preferences and feeding habits of the two species of *Ptychobarbus*, are needed to further explore the molecular mechanism underlying their adaptive differentiation and speciation.

## Supplementary Information

Supplementary Material 1

## Data Availability

The datasets generated during the current study are available in the National Center for Biotechnology Information database (No. PRJNA1315248).

## References

[1] Liu JQ, Wang YJ, Wang AL, Hideaki O, Abbott RJ. Radiation and diversification within the Ligularia-Cremanthodium-Parasenecio complex (Asteraceae) triggered by uplift of the Qinghai-Tibetan plateau. Mol Phylogenet Evol. 2006;38(1):31–49. 16290033 10.1016/j.ympev.2005.09.010

[2] Zhang DR, Chen MY, Murphy RW, Che J, Pang JF, Hu JS, Luo J, Wu SJ, Ye H, Zhang YP. Genealogy and palaeodrainage basins in Yunnan province: phylogeography of the Yunnan spiny frog, Nanorana yunnanensis (Dicroglossidae). Mol Ecol. 2010;19(16):3406–20. 20666999 10.1111/j.1365-294X.2010.04747.x

[3] Wang J, Raisbeck G, Xu JB, Yiou F, Bai SB. 10Be chronology of quaternary glaciation at the Southern end of the Shaluli mountains in the Southeast of the Qinghai Tibet plateau. China Sci. 2006;36(8):706–12.

[4] Zhou SZ, Xu LB, Cui JX, Zhang XW, Zhao JD. Quaternary geomorphic development and environmental evolution of the Sululi mountains. Sci Bull. 2004;49(23):2480–4.

[5] Huang SY, Chen YY. Phylogenetic relationship and zoogeographical analysis of *Ptychobarbus chungtienensis* and *P. kaznakovi*. Acta Zool Sin. 1986;11(1):100–7.

[6] Zhang CG, Yang JX, Zhao YH, Pan XF. Fish in Jinsha river basin. Beijing: Science; 2019.

[7] Wu YF, Wu CZ. Qinghai Tibet plateau fish. Chengdu: Sichuan Science and Technology; 1992.

[8] Jiao Y, Chen Y, Wroblewski J. An application of the composite risk assessment method in assessing fisheries stock status. Fish Res. 2005;72(2–3):173–83.

[9] Yan F, Zhou W, Zhao H, Yuan Z, Wang Y, Jiang K, Jin J, Murphy RW, Che J, Zhang Y. Geological events play a larger role than pleistocene Climatic fluctuations in driving the genetic structure of Quasipaa boulengeri (Anura: Dicroglossidae). Mol Ecol. 2013;22(4):1120–33. 23216961 10.1111/mec.12153

[10] Catchen J, Hohenlohe PA, Bassham S, Amores A, Cresko WA. Stacks: an analysis tool set for population genomics. Mol Ecol. 2013;22(11):3124–40. 23701397 10.1111/mec.12354 PMC3936987

[11] Chen S, Zhou Y, Chen Y, Gu J. Fastp: an ultra-fast all-in-one FASTQ preprocessor. Bioinf (Oxford England). 2018;34(17):i884–90. 10.1093/bioinformatics/bty560 PMC6129281 30423086

[12] Li H, Durbin R. Fast and accurate short read alignment with Burrows-Wheeler transform. Bioinf (Oxford England). 2009;25(14):1754–60. 10.1093/bioinformatics/btp324 PMC2705234 19451168

[13] Xiao S, Mou Z, Fan D, Zhou H, Zou M, Zou Y, Zhou C, Yang R, Liu J, Zhu S, et al. Genome of tetraploid fish *Schizothorax o’connori *provides insights into early Re-diploidization and High-Altitude adaptation. iScience. 2020;23(9):101497. 32905880 10.1016/j.isci.2020.101497 PMC7486454

[14] Okonechnikov K, Conesa A, García-Alcalde F. Qualimap 2: advanced multi-sample quality control for high-throughput sequencing data. Bioinf (Oxford England). 2016;32(2):292–4. 10.1093/bioinformatics/btv566 PMC4708105 26428292

[15] Heldenbrand JR, Baheti S, Bockol MA, Drucker TM, Hart SN, Hudson ME, Iyer RK, Kalmbach MT, Kendig KI, Klee EW, et al. Correction to: Recommendations for performance optimizations when using GATK3.8 and GATK4. BMC Bioinformatics. 2019;20(1):722. 31847808 10.1186/s12859-019-3277-4 PMC6918610

[16] Danecek P, Auton A, Abecasis G, Albers CA, Banks E, DePristo MA, Handsaker RE, Lunter G, Marth GT, Sherry ST, et al. The variant call format and vcftools. Bioinf (Oxford England). 2011;27(15):2156–8. 10.1093/bioinformatics/btr330 PMC3137218 21653522

[17] Excoffier L, Lischer HE. Arlequin suite ver 3.5: a new series of programs to perform population genetics analyses under Linux and windows. Mol Ecol Resour. 2010;10(3):564–7. 21565059 10.1111/j.1755-0998.2010.02847.x

[18] Olivier DR. Getting a tree fast: neighbor Joining, FastME, and distance-based methods. Curr Protocols Bioinf. 2006;6(6):631–8. 10.1002/0471250953.bi0603s15 18428768

[19] Vilella AJ, Severin J, Ureta-Vidal A, Heng L, Durbin R, Birney E. EnsemblCompara genetrees: Complete, duplication-aware phylogenetic trees in vertebrates. Genome Res. 2009;19(2):327–35. 19029536 10.1101/gr.073585.107 PMC2652215

[20] Noma H, Nagashima K, Maruo K, Gosho M, Furukawa TA. Bartlett-type corrections and bootstrap adjustments of likelihood-based inference methods for network meta-analysis. Stat Med. 2018;37(7):1178–90. 29250816 10.1002/sim.7578

[21] Purcell S, Neale B, Todd-Brown K, Thomas L, Ferreira MA, Bender D, Maller J, Sklar P, de Bakker PI, Daly MJ, et al. PLINK: a tool set for whole-genome association and population-based linkage analyses. Am J Hum Genet. 2007;81(3):559–75. 17701901 10.1086/519795 PMC1950838

[22] Alexander DH, Novembre J, Lange K. Fast model-based Estimation of ancestry in unrelated individuals. Genome Res. 2009;19(9):1655–64. 19648217 10.1101/gr.094052.109 PMC2752134

[23] Terhorst J, Kamm JA, Song YS. Robust and scalable inference of population history from hundreds of unphased whole genomes. Nat Genet. 2017;49(2):303–9. 28024154 10.1038/ng.3748 PMC5470542

[24] Li Z, Zhu FY, Liu MD, Wang Q, Liu SP. Age structure and growth characteristics of *Ptychobarbus Kaznakovi* in the upper reaches of the Nu river. Freshw Fish Sect. 2019;49(4):8.

[25] Pickrell JK, Pritchard JK. Inference of population splits and mixtures from genome-wide allele frequency data. PLoS Genet. 2012;8(11):e1002967. 23166502 10.1371/journal.pgen.1002967 PMC3499260

[26] Wang J, Hu Z, Liao X, Wang Z, Li W, Zhang P, Cheng H, Wang Q, Bhat JA, Wang H, et al. Whole-genome resequencing reveals signature of local adaptation and divergence in wild soybean. Evol Appl. 2022;15(11):1820–33. 36426120 10.1111/eva.13480 PMC9679240

[27] Zhao X, Guo Y, Kang L, Yin C, Bi A, Xu D, Zhang Z, Zhang J, Yang X, Xu J, et al. Population genomics unravels the holocene history of bread wheat and its relatives. Nat Plants. 2023;9(3):403–19. 36928772 10.1038/s41477-023-01367-3

[28] Qiu Q, Wang L, Wang K, Yang Y, Ma T, Wang Z, Zhang X, Ni Z, Hou F, Long R, et al. Yak whole-genome resequencing reveals domestication signatures and prehistoric population expansions. Nat Commun. 2015;6:10283. 26691338 10.1038/ncomms10283 PMC4703879

[29] Hufford MB, Xu X, van Heerwaarden J, Pyhäjärvi T, Chia JM, Cartwright RA, Elshire RJ, Glaubitz JC, Guill KE, Kaeppler SM, et al. Comparative population genomics of maize domestication and improvement. Nat Genet. 2012;44(7):808–11. 22660546 10.1038/ng.2309 PMC5531767

[30] Du J, Yuan Z, Ma Z, Song J, Xie X, Chen Y. KEGG-PATH: Kyoto encyclopedia of genes and genomes-based pathway analysis using a path analysis model. Mol Biosyst. 2014;10(9):2441–7. 24994036 10.1039/c4mb00287c

[31] Kanehisa M. Toward Understanding the origin and evolution of cellular organisms. Protein Science: Publication Protein Soc. 2019;28(11):1947–51. 10.1002/pro.3715 PMC6798127 31441146

[32] Guo YS, Sun ZY, He XH, Jin W, Chen YL. Sichuan fish primary color atlas II. Beijing: Science; 2021.

[33] Chen YX. Hengduan mountain fish. Bejing: Science; 1998.

[34] Ding RH. Sichuan ichthyology. Chengdu: Sichuan Science and Technology; 1994.

[35] Bradic M, Beerli P, Francisco JGL, Esquivel-Bobadilla S, Borowsky RL. Gene flow and population structure in the Mexican blind cavefish complex (*Astyanax mexicanus*). BMC Evol Biol. 2012;12(1):9. 22269119 10.1186/1471-2148-12-9 PMC3282648

[36] Dong WW. Study on morphological characteristics and population genetics of *Xenophysogobio boulengeri* and *Xenophysogobio**nudicorpa*. Chongqing: Southwest University; 2019.

[37] Sun YM. Study on species differentiation of rhinogobio. Shandong: Shandong University; 2008.

[38] Spicer RA, Farnsworth A, Su T. Cenozoic topography, monsoons and biodiversity conservation within the Tibetan region: an evolving story. Plant Divers. 2020;42(4):229–54. 33094197 10.1016/j.pld.2020.06.011 PMC7567768

[39] Spicer RA, Su T, Valdes PJ, Farnsworth A, Zhou ZK. The topographic evolution of the Tibetan region as revealed by palaeontology. J Palaeobiodiversity Palaeoenvironments. 2020;101:213–43.

[40] Spicer RA, Su T, Valdes PJ, Farnsworth A, Wu FX, Shi G, Spicer TEV, Zhou ZK. Why ‘the uplift of the Tibetan plateau’ is a myth. Natl Sci Rev. 2020;8(1):nwaa091. 34691550 10.1093/nsr/nwaa091 PMC8288424

[41] He K, Jiang XL. Sky Islands of Southwest China. I: an overview of phylogeographic patterns. Chin Sci Bull. 2014;59(7):1–13.

[42] Hughes CE, Atchison GW. The ubiquity of alpine plant radiations: from the Andes to the Hengduan mountains. New Phytol. 2015;207(2):275. 25605002 10.1111/nph.13230

[43] Luo D, Xu B, Li ZM, Sun H. The ‘Ward Line-Mekong-Salween divide’ is an important floristic boundary between the Eastern himalaya and Hengduan mountains: evidence from the phylogeographical structure of subnival herbs marmoritis complanatum (Lamiaceae). Bot J Linn Soc. 2017;185(4):482–96.

[44] Yang L, Wang Y, Sun N, Chen J, He S. Genomic and functional evidence reveals convergent evolution in fishes on the Tibetan plateau. Mol Ecol. 2021;30(22):5752–64. 34516715 10.1111/mec.16171

[45] Liu JQ, Duan Y, Hao G, Ge X, Sun H. Evolutionary history and underlying adaptation of alpine plants on the Qinghai–Tibet plateau. J Syst Evol. 2014;52:241–9.

[46] Wen J, Zhang JQ, Nie ZL, Zhong Y, Sun H. Evolutionary diversifications of plants on the Qinghai-Tibetan plateau. Front Genet. 2014;5:4. 24575120 10.3389/fgene.2014.00004 PMC3921583

[47] Favre A, Pckert M, Pauls SU, Jhnig SC, Uhl D, Michalak I, Alexandra NMR. The role of the uplift of the Qinghai-Tibetan plateau for the evolution of Tibetan biotas. Biol Rev Camb Philos Soc. 2015;90(1):236–53. 24784793 10.1111/brv.12107

[48] Muellner-Riehl AN. Mountains as evolutionary arenas: Patterns, emerging Approaches, paradigm Shifts, and their implications for plant phylogeographic research in the Tibeto-Himalayan region. Front Plant Sci. 2019;10:195. 30936883 10.3389/fpls.2019.00195 PMC6431670

[49] Wu S, Wang Y, Wang Z, Shrestha N, Liu J. Species divergence with gene flow and hybrid speciation on the Qinghai-Tibet plateau. New Phytol. 2022;234(2):392–404. 35020198 10.1111/nph.17956

[50] Liu D, Hou F, Liu Q, Zhang X, Yan T, Song Z. Strong population structure of schizopygopsis Chengi and the origin of S. Chengi baoxingensis revealed by MtDNA and microsatellite markers. Genetica. 2015;143(1):73–84. 25572029 10.1007/s10709-015-9815-8

[51] Qi DL, Guo SC, Zhao XQ, Yang J, Tangi WJ. Genetic diversity and historical population structure of *S**chizopygopsis** pylzovi *(Teleostei: Cyprinidae) in the Qinghai-Tibetan plateau. Freshw Biol. 2010;52(6):1090–104.

[52] Li ZH, Liu ZL, Wang ML, Qian ZQ, Zhao P, Zhu J, Yang YX, Yan XH, Li YJ, Zhao GF. A review on studies of speciation in the presence of gene flow: evolution of reproductive isolation. Biodivers Sci. 2014;22(1):88–96.

[53] Marianne E, Rui F, Zachariah G, Andrew H. Factors influencing progress toward ecological Speciation. Int J Ecol. 2012;2012(1):1–7.

[54] Patrick B. Speciation of the genus barbus in the North mediterranean basin: recent advances from biochemical genetics. Biol Conserv. 1995;72(2):237–49.

[55] He SP, Liu HZ, Chen YY, Masayuki K, Tsuneo N, Zhong Y. Phylogeny of Cyprinidae based on *cytochrome b* gene sequence (ichthya: Cypriniformes). Sci China Ser C: Life Sci. 2004;34(1):96–104.

[56] Zheng LP, Yang JX, Chen XY. Molecular phylogeny and systematics of the barbinae (Teleostei: Cyprinidae) in China inferred from mitochondrial DNA sequences. ;*[ 1 ] Chinese acad Sci, Kunming Inst Zool, state key lab genet resources & Evolut, 32 Jiaochang Donglu, Kunming 650223, Yunnan, peoples R China [ 2 ]*. Chin Acad Sci Southeast Asia Biodivers Res Inst Yezin. 2016;05282:68:250–9.

[57] Wang JF, Pan YZ, Gong X, Chiang YC, KURODA C. Chloroplast DNA variation and phylogeography of ligularia tongolensis (Asteraceae), a species endemic to the Hengduan mountains region of China. J Syst Evol. 2011;49(2):108–19.

[58] Guo-Dong LI, Yue LL, Sun H, Qian ZG. Phylogeography of *Cyananthus**delavayi* (Campanulaceae) in Hengduan mountains inferred from variation in nuclear and Chloroplast DNA sequences. J Syst Evol. 2012;50(4):11.

[59] Lu B, Zheng YC, Murphy RW, Zeng XM. Coalescence patterns of endemic Tibetan species of stream salamanders (Hynobiidae:Batrachuperus). Mol Ecol. 2012;21(13):3308–24. 22571598 10.1111/j.1365-294X.2012.05606.x

[60] Chen W, Liu S, Liu Y, Hao H, Zeng B, Chen S, Peng H, Yue B, Zhang X. Phylogeography of the large White-bellied rat niviventer excelsior suggests the influence of pleistocene glaciations in the Hengduan mountains %J. Zoolog Sci. 2010;27(6):487–93. 20528155 10.2108/zsj.27.487

[61] Liu FW, Ma LL, Yang CZ, Tu FY, Zhang XY. Taxonomic status of *Tetraophasis obscurus* and *Tetraophasis szechenyii *(Aves: galliformes: Phasianidae) based on the complete mitochondrial genome. Zoolog Sci. 2014;31(3):160. 24601778 10.2108/zsj.31.160

[62] Chen WC, Liu SY, Liu Y, Hao HB, Zeng B, Chen SD, Peng HY, Yue BS, Zhang XY. Phylogeography of the large White-bellied rat niviventer excelsior suggests the influence of pleistocene glaciations in the Hengduan mountains. Zoolog Sci. 2010;27(6):487–93. 20528155 10.2108/zsj.27.487

[63] Ma BS, Wei KJ, Zhao TY, Pei FC, Huo B. Research progress on phylogeny and plateau adaptability of schizothorax. Lake Sci. 2023;35(3):808–20.

[64] Li JD, Song G, Liu NF, Chang YB, Bao XK. Deep south-north genetic divergence in godlewski’s bunting (*Emberiza godlewskii*) related to uplift of the Qinghai-Tibet plateau and habitat preferences. BMC Evol Biol. 2019;19(1):161. 31370783 10.1186/s12862-019-1487-z PMC6676563

[65] Chen YJ, Zhu L, Wu QN, Hu CC, Qu YF, Ji X. Geological and climatic influences on population differentiation of the Phrynocephalus vlangalii species complex (Sauria: Agamidae) in the northern Qinghai-Tibet Plateau. Mol Biol Evol. 2022;169(Suppl C):107394. 10.1016/j.ympev.2022.107394 35045310

[66] Wei SC, Li ZT, Momigliano P, Fu C, Wu H, Merilä JH. The roles of climate, geography and natural selection as drivers of genetic and phenotypic differentiation in a widespread amphibian Hyla annectans (Anura: Hylidae). Mol Ecol. 2020;29(19):3667–83. 32762086 10.1111/mec.15584

[67] David KT. Global gradients in the distribution of animal polyploids. Proceedings of the National Academy of Sciences of the United States of America. 2022;119(48):e2214070119. 36409908 10.1073/pnas.2214070119 PMC9860298

[68] Li X, Yang K, Tong L, Hou F, Liu Q, Li J, Lu Y, Song Z. Phylogeography of *Schizopygopsis malacanthus Herzenstein* (Cypriniformes, Cyprinidae) in relation to the tectonic events and quaternary Climatic oscillations in the Shaluli mountains region. Zoology. 2020;143:125835. 32949977 10.1016/j.zool.2020.125835

[69] Li X, Wang M, Zou M, Guan X, Xu S, Chen W, Wang C, Chen Y, He S, Guo B. Recent and recurrent autopolyploidization fueled diversification of snow sarp on the Tibetan Plateau. Mol Biol Evol. 2024;41(11):msae221. 39437268 10.1093/molbev/msae221 PMC11542630

[70] Shi YF, Li JJ, Li BY, Yao TD, Wang SM, Li SJ, Cui ZJ, Wang FB, Pan BT, Fang XM. Uplift of the Tibetan plateau in the late cenozoic and environmental changes in East Asia. Acta Geogr Sin. 1999;5(1):12–22.

[71] Xu LB. Quaternary glacier and geomorphic evolution in the Shaluli mountain area. Lanzhou: Lanzhou University; 2005.

[72] Xu LB, Zhou SZ. The impact of pleistocene glaciation and Southwest monsoon fluctuations on glaciation during the last glacial period in Shaluli mountain. Quatern Res. 2005;6(5):620–9.

[73] Fang XM, Chen FB. Ganzi loess and the evolution of the cryosphere on the Qinghai Tibet Plateau. Glacier Permafr. 1996;18(3):193–200.

[74] Outi S, Martin L, Juha M. Ecological genomics of local adaptation. Nat Rev Genet. 2013;14(11):807–20. 24136507 10.1038/nrg3522

[75] Tiffin P, Ross-Ibarra J. Advances and limits of using population genetics to understand local adaptation. Trends Ecol Evol. 2014;29(10):673–80. 25454508 10.1016/j.tree.2014.10.004

[76] Hu HY, Yang YZ, Li A, Zheng ZY, Zhang J, Liu JQ. Genomic divergence of Stellera Chamaejasme through local selection across the Qinghai-Tibet plateau and Northern China. Mol Ecol. 2022;31(18):4782–96. 35869662 10.1111/mec.16622

[77] Feng S, Wan W, Li Y, Wang D, Ren G, Ma T, Ru D. Transcriptome-based analyses of adaptive divergence between two closely related Spruce species on the Qinghai-Tibet plateau and adjacent regions. Mol Ecol. 2023;32(2):476–91. 36320185 10.1111/mec.16758

[78] Hallet B, Molnar P. Distorted drainage basins as markers of crustal strain East of the himalaya. J Geophys Res Biogeosciences. 2001;106(B7):13697–709.

[79] Tong C, Li M, Zhao K. Transcriptomic signature of rapidly evolving immune genes in a Highland fish. Fish Shellfish Immunol. 2019;97:587–92. 31891809 10.1016/j.fsi.2019.12.082

[80] Tong C, Fei T, Zhang CF, Zhao K. Comprehensive transcriptomic analysis of Tibetan schizothoracinae fish gymnocypris Przewalskii reveals how it adapts to a high altitude aquatic life. BMC Evol Biol. 2017;17(1):74. 28274203 10.1186/s12862-017-0925-z PMC5343388

[81] Macfadyen EJ, Williamson CE, Grad G, Lowery M, Mitchell DL. Molecular response to climate change: temperature dependence of UV-induced DNA damage and repair in the freshwater crustacean daphnia pulicaria. Glob Change Biol. 2010;10(4):408–16.

[82] Morison SA, Cramp RL, Alton LA, Franklin CE. Cooler temperatures slow the repair of DNA damage in tadpoles exposed to ultraviolet radiation: implications for amphibian declines at high altitude. Glob Change Biol. 2020;26(3):1225–34. 10.1111/gcb.14837 31518484

[83] Albarracín VH, Pathak GP, Douki T, Cadet J, Borsarelli CD, Gärtner W, Farias ME. Extremophilic acinetobacter strains from High-Altitude lakes in Argentinean puna: remarkable UV-B resistance and efficient DNA damage repair. Origins Life Evol Biospheres. 2012;42(2–3):201–21. 10.1007/s11084-012-9276-3 22644565

[84] Zhao SY, Chen LY, John KM, Hu GW, Wang QF. Genetic adaptation of giant *Lobelias* (*Lobelia**aberdarica* and *Lobelia**telekii*) to different altitudes in East African mountains. Front Plant Sci. 2016;7:488. 27148313 10.3389/fpls.2016.00488 PMC4828460

[85] Yang YZ, Wang LZ, Han J, Tang XL, Ma M, Wang K, Zhang X, Ren Q, Chen Q, Qiu Q. Comparative transcriptomic analysis revealed adaptation mechanism of *Phrynocephalus erythrurus*, the highest altitude Lizard living in the Qinghai-Tibet Plateau. BMC Evol Biol*.* 2015;15(1):101. 10.1186/s12862-015-0371-8 PMC4450828 26031664

[86] Zhou CW, Xiao SJ, Liu YC, Mou ZB, Liu HP. Comprehensive transcriptome data for endemic schizothoracinae fish in the Tibetan plateau. Sci Data. 2020;7(1):28. 31964888 10.1038/s41597-020-0361-6 PMC6972879

[87] Gao K, He Z, Xiong JX, Chen QQ, Lai BL, Liu F, Chen P, Chen MQ, Luo WJ, Huang JJ, et al. Population structure and adaptability analysis of *Schizothorax**o’connori* based on whole-genome resequencing. BMC Genomics. 2024;25(1):145. 38321406 10.1186/s12864-024-09975-9 PMC10845765

[88] Yuan DY. Study on genetic basis of plateau adaptability and low temperature tolerance of trilophysa bleekeri. Chongqing: Southwest University; 2021.

[89] Wei K, Silva-Arias GA, Tellier A. Selective sweeps linked to the colonization of novel habitats and Climatic changes in a wild tomato species. New Phytol. 2023;237(5):1908–21. 36419182 10.1111/nph.18634

[90] Simpson GG. Principles of animal taxonomy. New York: Columbia University; 1961. 10.1126/science.133.3464.1589 17781120

[91] Conroy CJ, Cook JA. Phylogeography of a post-glacial colonizer: microtus longicaudus (Rodentia: muridae). Mol Ecol. 2010;9(2):165–75. 10.1046/j.1365-294x.2000.00846.x 10672160

[92] Orr HA, Turelli M. The evolution of postzygotic isolation: accumulating Dobzhansky-Muller incompatibilities. Evolution. 2001;55(6):1085–94. 11475044 10.1111/j.0014-3820.2001.tb00628.x

[93] Wang Y, Yang LD, Zhou K, Zhang YP, Song ZB, He SP. Evidence for adaptation to the Tibetan plateau inferred from Tibetan loach transcriptomes. Genome Biol Evol. 2015;7(11):2970–82. 26454018 10.1093/gbe/evv192 PMC5635588

[94] Qiu QW, Lizhong, Wang K, Yang YZ, Ma T, Wang ZF, Zhang X, Ni ZQ, Hou FJ, Long RJ. Yak whole-genome resequencing reveals domestication signatures and prehistoric population expansions. Nat Commun. 2015;6:10283. 26691338 10.1038/ncomms10283 PMC4703879

[95] Blanco AM. Hypothalamic- and Pituitary-Derived growth and reproductive hormones and the control of energy balance in fish. Gen Comp Endocrinol. 2019;287:113322. 31738909 10.1016/j.ygcen.2019.113322

[96] Sheridan MA. Coordinate regulation of feeding, metabolism, and growth: perspectives from studies in fish. Gen Comp Endocrinol. 2021;312:113873. 34329604 10.1016/j.ygcen.2021.113873

[97] Holzem M, Boutros M, Holstein TW. The origin and evolution of Wnt signalling. Nat Rev Genet. 2024;25(7):500–12. 38374446 10.1038/s41576-024-00699-w

[98] He JY, He Z, Yang DY, Ma ZJ, Chen HJ, Zhang Q, Deng FQ, Ye LJ, Pu Y, Zhang MW, et al. Genetic variation in *Schizothorax**Kozlovi**Nikolsk*y in the upper reaches of the Chinese Yangtze river based on genotyping for simplified genome sequencing. Animals. 2022;12(17):2181. 36077902 10.3390/ani12172181 PMC9454844

[99] Miao D, Ren J, Jia Y, Jia Y, Li Y, Huang H, Gao, RJCc. CCS s: PAX1 represses canonical Wnt signaling pathway and plays dual roles during endoderm differentiation. Cell Commun Signal. 2024;22(1):242. 38664733 10.1186/s12964-024-01629-3 PMC11046865

