# Supplementary material for: Genome-wide insights into adaptive divergence, historical demography, and habitat suitability of Ptychobarbus Kaznakovi and P. leptosomus
Source: BMC Genomics. 2026 Feb 25;27:331. doi: 10.1186/s12864-026-12664-4 (PMC13041509; doi:10.1186/s12864-026-12664-4)
Supplement: Supplementary file 1 — Supplementary Material 1 [file 12864_2026_12664_MOESM1_ESM.docx]

**Supplementary tables:**

**Table S1. Measurement indexes of morphological characteristics of *Ptychobarbus kaznakovi and P. leptosomus***

| Number | Moprhological trait | Detailed description |
| --- | --- | --- |
| D1 | A-B | Rostral to posterior margin of mandible |
| D2 | A-C | Rostral to occipital posterior |
| D3 | A-E | Rostral end to starting point of pectoral fin base |
| D4 | A-G | From rostral end to starting point of ventral fin base |
| D5 | B-C | Posterior margin of mandible to occipital posterior |
| D6 | B-D | Posterior margin of mandible to the end of mandible |
| D7 | C-D | Posterior occipital to distal mandible |
| D8 | C-E | Occipital posterior to origin of pectoral fin base |
| D9 | C-F | Occipital posterior to dorsal fin base starting point |
| D10 | C-G | Occipital posterior to the starting point of ventral fin base |
| D11 | E-F | Starting point of pectoral fin base to starting point of dorsal fin base |
| D12 | F-G | Starting point of dorsal fin base to starting point of abdominal fin base |
| D13 | F-H | From the beginning of dorsal fin base to the end of dorsal fin base |
| D14 | F-I | Dorsal fin origin to gluteal fin base origin |
| D15 | G-H | Starting point of ventral fin base to end of dorsal fin base |
| D16 | G-I | Starting point of ventral fin base to starting point of gluteal fin base |
| D17 | H-I | From the base of dorsal fin to the base of gluteal fin |
| D18 | H-J | From the base end of dorsal fin to the starting point of the back of caudal fin |
| D19 | H-K | Base end of dorsal fin to abdominal origin of caudal fin |
| D20 | I-J | From the base of gluteal fin to the back of caudal fin |
| D21 | I-K | From the base of gluteal fin to the abdomen of caudal fin |
| D22 | J-K | Starting point at the back of caudal fin to starting point at the abdomen of caudal fin |
| D23 | Head length | Rostral end to posterior edge of operculum |
| D24 | Snout length | Rostral to anterior orbital margin |
| D25 | Eye diameter | Anterior to posterior orbital margin |
| D26 | Distance between eyes | Minimum width between bilateral superior orbital margins |
| D27 | Length of head behind eye | Distance from the posterior edge of the orbit to the posterior edge of the operculum |
| D28 | Body height | Maximum height of fish |
| D29 | Body width | Maximum width of fish body |
| D30 | Dorsal fin length |  |
| D31 | Pectoral fin length |  |
| D32 | Ventral fin length |  |
| D33 | Anal fin length |  |
| D34 | Caudal fin length |  |
| D35 | Caudal stalk length | **From the base of the gluteal fin to the end of the spine** |
| D36 | Caudal stalk height | **Minimum height of tail handle** |
| D37 | Caudal stalk width | **Narrowest width of tail handle** |
| D38 | Body length | **Horizontal distance from the rostral end to the last caudal vertebra** |

**Table S2. Comparison of traditional morphological data between *Ptychobarbus kaznakovi* and *P. leptosomus***

| Characteristics | *Ptychobarbus kaznakovi* | *P. leptosomus* |
| --- | --- | --- |
| Body length/height | 4.34~5.53 | 4.01~5.70 |
| Body length/head length | 3.90~4.92 | 3.54~4.95 |
| Body length/tail handle length | 5.98~7.62 | 6.13~7.74 |
| Body length/tail handle height | 11.10~13.13 | 11.38~14.53 |
| Head length/snout length | 2.90~3.70 | 2.72~4.11 |
| Head length/eye diameter | 4.51~6.85 | 4.03~6.55 |
| Tail handle length/tail handle height | 1.69~2.61 | 1.42~2.31 |

Supplementary figure:


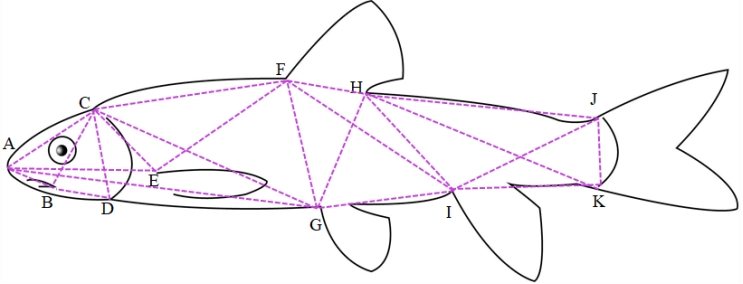


**Figure S1. Shape measurement outline**
